# Lack of whey acidic protein (WAP) four-disulfide core domain protease inhibitor 2 (WFDC2) causes neonatal death from respiratory failure in mice

**DOI:** 10.1242/dmm.040139

**Published:** 2019-11-12

**Authors:** Kuniko Nakajima, Michio Ono, Uroš Radović, Selma Dizdarević, Shin-ichi Tomizawa, Kazushige Kuroha, Go Nagamatsu, Ikue Hoshi, Risa Matsunaga, Takayuki Shirakawa, Takeyuki Kurosawa, Yasunari Miyazaki, Masahide Seki, Yutaka Suzuki, Haruhiko Koseki, Masataka Nakamura, Toshio Suda, Kazuyuki Ohbo

**Affiliations:** 1 Department of Histology and Cell Biology, Yokohama City University, School of Medicine, 3-9 Fukuura, Kanazawa-ku, Yokohama, 236-0004, Japan; 2 Department of Stem Cell Biology, Kyushu University, Faculty of Medical Sciences, 3-1-1, Maidashi, Higashi-ku, Fukuoka City, 812-8582, Japan; 3 Department of Respiratory Medicine, Toho University, School of Medicine, 5-21-16, Ohmorinishi, Ohta-ku, Tokyo, Japan; 4 Department of Respiratory Medicine, Tokyo Medical and Dental University, 1-5-45, Yushima, Bunkyo-ku, Tokyo, 113-8510, Japan; 5 Department of Computational Biology and Medical Sciences, Graduate School of Frontier Sciences, The University of Tokyo, Chiba, 277-8562, Japan; 6 Laboratory for Developmental Genetics, RIKEN Center for Integrative Medical Sciences, 1-7-22, Tsurumi-ku, Yokohama, 230-0045, Japan; 7 Human Gene Sciences Center, Tokyo Medical and Dental University, 1-5-45, Yushima, Bunkyo-ku, Tokyo, 113-8510, Japan; 8 Cancer Science Institute of Singapore, National Singapore University Centre for Translational Medicine, 14 Medical Drive, #12-01, Singapore 117599; 9 International Research Center for Medical Sciences, Kumamoto University, 2-2-1 Honjo, Chuo-ku, Kumamoto, 860-0811, Japan

**Keywords:** Cilia, Surfactant, Atelectasis, Respiratory disease

## Abstract

Respiratory failure is a life-threatening problem for pre-term and term infants, yet many causes remain unknown. Here, we present evidence that whey acidic protein (WAP) four-disulfide core domain protease inhibitor 2 (Wfdc2), a protease inhibitor previously unrecognized in respiratory disease, may be a causal factor in infant respiratory failure. *Wfdc2* transcripts are detected in the embryonic lung and analysis of a *Wfdc2-GFP* knock-in mouse line shows that both basal and club cells, and type II alveolar epithelial cells (AECIIs), express *Wfdc2* neonatally. *Wfdc2*-null-mutant mice display progressive atelectasis after birth with a lethal phenotype. Mutant lungs have multiple defects, including impaired cilia and the absence of mature club cells from the tracheo-bronchial airways, and malformed lamellar bodies in AECIIs. RNA sequencing shows significant activation of a pro-inflammatory pathway, but with low-quantity infiltration of mononuclear cells in the lung. These data demonstrate that *Wfdc2* function is vitally important for lung aeration at birth and that gene deficiency likely causes failure of the lung mucosal barrier.

## Abstract (precis)

**Summary:** Wfdc2 is vitally important in preventing atelectasis and dysfunction of barrier mechanisms such as mucociliary clearance, intercellular junction formation and anti-inflammatory activity.

## INTRODUCTION

During the transition from fetal to neonatal life, the cardio-respiratory system undergoes a drastic and sudden change: placental circulation switches to a pulmonary circulation and the respiratory system goes from being fluid filled to air filled. Malfunctions of this switch can cause respiratory failure (Reuter et al., 2014), in particular in premature infants in whom the immature lung tissues have produced inadequate mucosal fluid (Reuter et al., 2014). The components of this fluid are produced by respiratory epithelial cells. There are at least eight different types of these cells, which form the primary barrier between air and lung tissue (Hogan et al., 2014; Montoro et al., 2018; Plasschaert et al., 2018; Rock and Hogan, 2011; Treutlein et al., 2014). In the airways, the epithelium consists of basal stem cells, and ciliated, club, goblet and neuroendocrine cells, as well as other rare cell types, including newly described ionocytes (Montoro et al., 2018; Plasschaert et al., 2018). The goblet and club cells secrete mucins and glycoproteins that have bactericidal and anti-inflammatory functions; the ciliated cells move the mucus and entrap particles out of the lungs; and the ionocytes, which highly express cystic fibrosis transmembrane conductance regulator (CFTR), likely play a role in regulating fluid secretion (Montoro et al., 2018; Plasschaert et al., 2018). In the alveolar region, the type II epithelial cells (AECIIs) produce important surfactants that consist of lipids [e.g. phosphatidylcholine (PC), phosphatidylglycerol (PG)] and proteins (e.g. SP-A, -B, -C, -D), which are stored in specialized organelles known as lamellar bodies (Chakraborty and Kotecha, 2013; Lopez-Rodriguez and Pérez-Gil, 2014; Olmeda et al., 2017; Pérez-Gil, 2008; Rooney et al., 1994; Whitsett and Alenghat, 2015). Surfactants form thin films that reduce surface tension, open up the alveolar space, and are critical for both preventing collapse of the airways (atelectasis) and for contributing to the host defense strategy (Chakraborty and Kotecha, 2013; Rooney et al., 1994; Whitsett and Alenghat, 2015; Whitsett et al., 2010).

In addition to mucins and surfactants, the epithelial cells of the lung secrete numerous proteases and protease inhibitors – such as serine proteases, matrix metalloproteinases (MMPs), serine protease inhibitors and tissue inhibitor of metalloproteinases (TIMPs) – that together form the protease/protease-inhibitor web (Löffek et al., 2011; Abboud, 2012; Meyer and Jaspers, 2015). These proteases and protease inhibitors are deeply involved in the pathophysiology of neonatal respiratory distress syndrome (RDS) as well as other lung diseases such as emphysema, cystic fibrosis, infection and pulmonary fibrosis (Meyer and Jaspers, 2015; Taggart et al., 2017). RDS is mainly caused by pulmonary surfactant deficiency in premature infants, and the lungs of RDS patients are diffusely atelectatic (Bhandari, 2014; Reuter et al., 2014). A subpopulation of RDS infants who show limited responses to surfactant may go on to develop chronic neonatal lung injury known as bronchopulmonary dysplasia (BPD), which involves failure of alveogenesis and vasculogenesis, and the risk of developing long-term morbidities (Bhandari, 2014; Dumpa and Bhandari, 2018). Inflammation is a cornerstone in the transition from RDS to BPD (Bhandari, 2014); its causes include hyperoxia and mechanical stress from ventilation, but proteases such as neutrophil elastase (NE) have also been shown to play crucial roles in the induction of IL-1α and TNFβ (Bhandari, 2014). However, the failure of α-antitrypsin therapy trials in human infants implies that other proteases and/or protease inhibitors may participate in the pathogenesis of both RDS and BPD, although a possibility remains that drug delivery efficiency to the affected areas might need to be improved (Beam et al., 2014).

One of the serine protease inhibitor families predicted to function in the innate immune system in the lung is the evolutionarily conserved group of whey acidic protein (WAP) four-disulfide core (FDC) proteins (WFDC protein family; Bingle and Vyakarnam, 2008). The WFDC signature – eight conserved cysteine residues linked by four disulfide bonds – is often found in antimicrobial and antifungal molecules (Jia et al., 2017; Scaletta et al., 2017), and inhibits protease activity (Bingle and Vyakarnam, 2008). Among the 14 WFDC mammalian genes (at least), many map to a cluster on chromosome 20q12-13 in humans (e.g. *SLPI*, *EPPIN* and *WFDC2*) or chromosome 2 in mice (Clauss et al., 2005; Bingle and Vyakarnam, 2008). This genomic region is amplified in many epithelial cancers, including breast, colon, lung, ovarian, pancreatic and stomach (Liu et al., 2013; O'Neal et al., 2013). In patients with serous and endometrioid epithelial ovarian carcinomas, a high serum WFDC2 level has been reported, and affords a good diagnostic and prognostic marker (Drapkin et al., 2005; Jia et al., 2017; Scaletta et al., 2017). Supporting the idea that the protein not only marks cancer cells but promotes cancerous growth, overexpression of *WFDC2* reportedly induces the proliferation and invasion of human ovarian cancer cells (Moore et al., 2014; Zhu et al., 2013). Similarly, in the respiratory system, the majority of adenocarcinomas and a percentage of squamous, small cell, and large cell carcinomas express high levels of WFDC2 protein, a marker again linked to a poor prognosis (Yamashita et al., 2012; Zhong et al., 2017). In a kidney fibrosis model, Wfdc2 reportedly suppresses the activity of serine proteases and metalloproteases (LeBleu et al., 2013). However, the physiological roles of Wfdc2 are just beginning to be revealed.

We show here that deleting *Wfdc2* in mice causes perinatal death due to respiratory failure soon after birth. *Wfdc2*-deficient neonatal mice have lung atelectasis of variable magnitude and at various locations. Our findings suggest that this phenotype is likely caused by damage to cilia, elimination of mature club cells in the tracheobronchial region and impairment of the processing of surfactants in AECIIs. Although histological analyses show low-quantity infiltration of mononuclear cells, RNA sequencing (RNA-seq) of lung samples showed significantly upregulated inflammatory networks, in postnatal but not in embryonic stages. Taken together, these results suggest that, *in* *vivo*, Wfdc2 plays critical roles in multiple aspects of lung function: it not only promotes mucociliary clearance but also confers anti-inflammatory activity and reduces surface tension. Therefore, Wfdc2 dysfunction may be a key factor in driving infant respiratory failure.

## RESULTS

### Expression of *Wfdc2* during development, and lung atelectasis and perinatal death in *Wfdc2* homozygous-null mutants

**Fig. 1.**
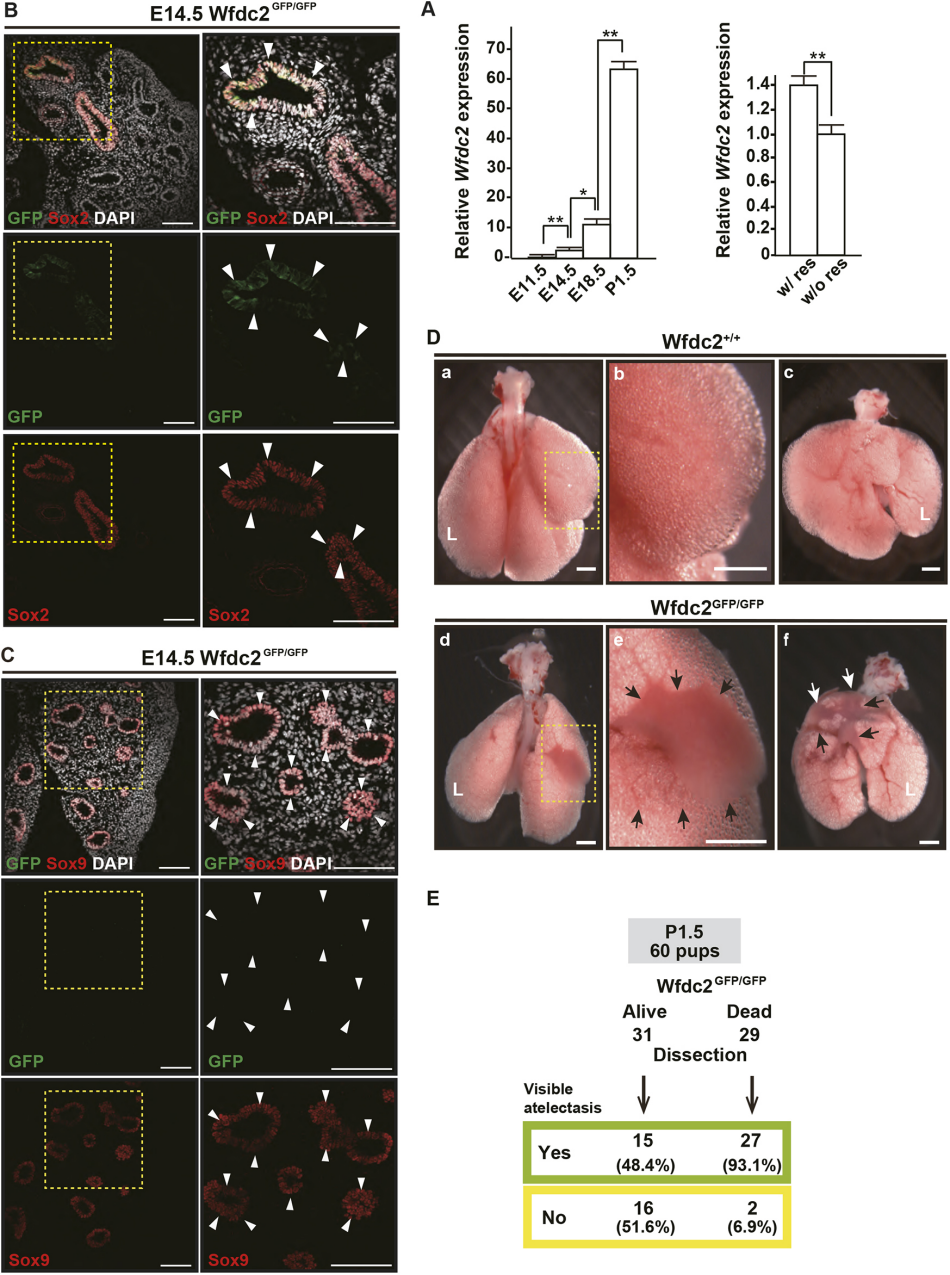
**Wfdc2 expression is detectable in the mouse proximal lung epithelium before birth and expression is upregulated after birth.** (A, left) Relative mRNA expression of *Wfdc2* during development. Data are shown as mean±s.e.m. (stages E11.5, *n*=4; E14.5-P1.5, *n*=2; *P<0.05; **P<0.01). (A, right) Induction of *Wfdc2* mRNA expression after cesarean section (CS), 1 day before due delivery. E18.5 embryos were obtained from pregnant mice by CS, resuscitated and processed for experimental samples 6-8 h after the CS (w/ res). As a control, other pregnant mice were sacrificed at the same point in time as the resuscitated fetus collection (w/o res). Data are shown as mean±s.e.m. (*n*=7 mice; **P<0.05). (B) Confocal images of the proximal region of a *Wfdc2*^GFP/GFP^ E14.5 lung. Yellow dotted boxes are enlarged in the right panel. Note that only the mesial parts (arrowheads) of the Sox2-positive region (red) express GFP (green). Nuclear DNA (DAPI) is shown in white. Scale bars: 100 µm. (C) Confocal images of the distal region of a *Wfdc2*^GFP/GFP^ E14.5 lung. The areas boxed by yellow dotted lines are enlarged in the right panels. There was no GFP (green) expression in Sox9-positive (red) regions (arrowheads). Nuclear DNA (DAPI) is shown in white. Scale bars: 100 µm. (D) Fresh lung specimens. (a,b,d,e) Dorsal view. (c,f) Frontal view. Both black and white arrows in e and f indicate areas affected by atelectasis. Boxed areas in panels a and d are enlarged in b and e, respectively. *Wfdc2*^+/+^ mice: a-c. *Wfdc2*^GFP/GFP^ mice: d-f. L, left lobe. Scale bars: 1 mm. (E) *Wfdc2*^GFP/GFP^ mice with atelectasis reveal high mortality. Out of 60 *Wfdc2-*deficient mice, 31 pups were alive and 29 pups died at between P0.5 and P1.5. Fifteen out of 31 live knockout mice showed atelectasis. However, 27 out of 29 dead pups displayed macroscopic atelectasis.

We initially measured *Wfdc2* RNA levels in the developing mouse lung at embryonic day (E)11.5, E14.5, E18.5 and postnatal day (P)1.5. Transcripts were already expressed at E11.5 and were strongly upregulated at P1.5 (Fig. 1A). Analysis of lungs 6-8 h after caesarean section at E18.5 revealed that mRNA expression rose significantly after respiration began (Fig. 1A). To track the lung epithelial cells that produce WFDC2, we generated knock-in mouse lines driving either *GFP* or *lacZ* from the *Wfdc2* locus (Fig. S1A-D). In agreement with the mRNA expression data, *Wfdc2-GFP* embryos showed signal from E14.5 (the pseudoglandular stage) in the proximal region of the bronchial tubes. The GFP-positive cells were located in the mesial part of the Sox2-positive proximal region (Fig. 1B), and few, if any, were seen in the distal, Sox9-positive, region (Fig. 1C).

Mice heterozygous for the GFP knock-in allele (*Wfdc2*^GFP/+^) were indistinguishable in lifespan and phenotype from wild-type littermates (Table 1). By contrast, the frequency of *Wfdc2*^GFP/GFP^ mice followed Mendel's laws until birth, but thereafter declined, and none survived post-weaning (Table 1). *Wfdc2*^GFP/GFP^ neonatal mice became cyanotic immediately after delivery (Movie 1), and all died within 10 days (Table 2), suggesting the cause of death was either heart failure or respiratory failure. Hearts from *Wfdc2*^GFP/GFP^ mice did not show any abnormality in volume or structure (Fig. S1E). *Wfdc2*^GFP/GFP^ lungs also did not show any abnormalities until birth (Fig. S1F). However, at P1.5, apparently collapsed regions could be detected (Fig. 1D), and this was confirmed by examining histological sections (Fig. S1G). The atelectasis was progressive (Fig. S1H). Among the 29 *Wfdc2-*deficient mice that died between P0.5 and P1.5, 93.1% had macroscopic atelectasis (Fig. 1E). By contrast, of the 31 surviving *Wfdc2*-deficient mice, only 48.3% had atelectasis (Fig. 1E). These results suggest that Wfdc2 is indispensable for functioning of the respiratory system after birth.

**Table 1.**
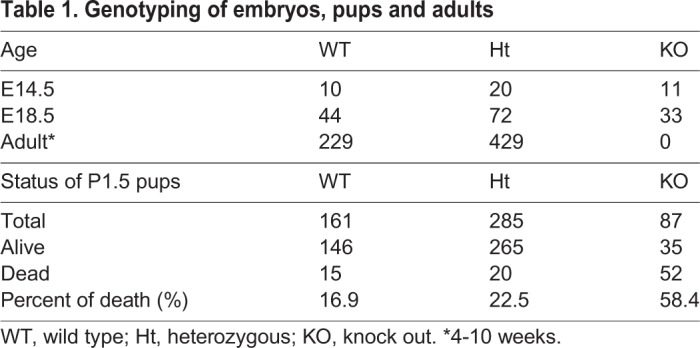
**Genotyping of embryos, pups and adults**

**Table 2.**
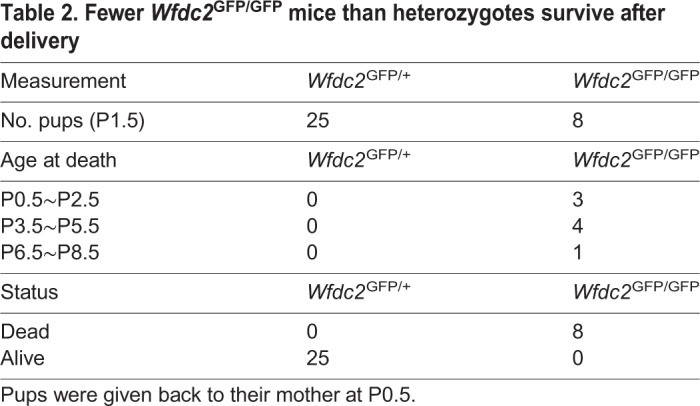
**Fewer *Wfdc2*^GFP/GFP^ mice than heterozygotes survive after delivery**

### Differentiation of Scgb1a1-positive club cells in the tracheobronchial region is impaired in *Wfdc2*-deficient mice

**Fig. 2.**
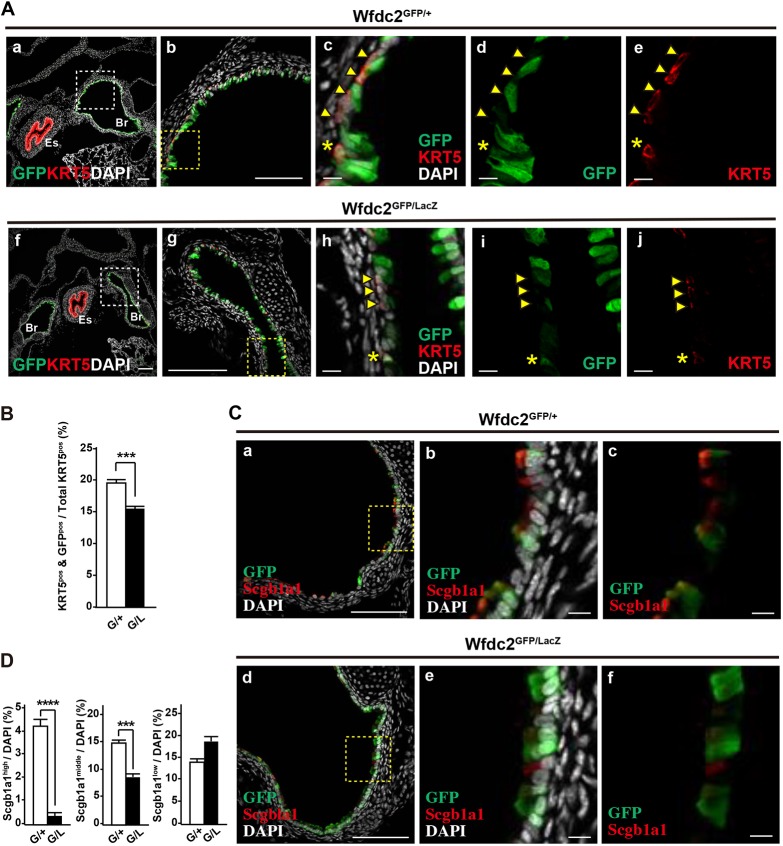
**Wfdc2 deficiency impairs differentiation of secretory cells.** (A) Representative confocal images of GFP^pos^ (green) and KRT5^pos^ (red) cells in the P1.5 bronchus. White dotted boxes in panels a and f are enlarged in b and g, respectively. Yellow dotted boxes in panels b and g are enlarged in c-e and h-j, respectively. Arrowheads indicate KRT5^pos^/GFP^neg^ basal cells. Asterisks indicate KRT5^pos^/GFP^pos^ basal cells. Nuclear DNA (DAPI) is shown in white. Es, esophagus; Br, main bronchus. Scale bars: 100 µm (a,b,f,g); 10 µm (c-e,h-j). (B) Quantification of GFP^pos^/KRT5^pos^ double-positive cells among total KRT5^pos^ cells in littermates of *Wfdc2*^GFP/+^ (G/+) and *Wfdc2*^GFP/LacZ^ (G/L). Data are shown as mean±s.e.m. (*n*=3 mice; ***P<0.005). *P*-values were calculated as compared with controls. (C) Representative confocal images of GFP^pos^ (green) and Scgb1a1^pos^ (red) cells in the P1.5 bronchus. Nuclear DNA (DAPI) is shown in white. Scale bars: 100 µm (a,d); 10 µm (b,c,e,f). (D) Quantification of Scgb1a1^high^, Scgb1a1^middle^ and Scgb1a1^low^ cells among total (DAPI^pos^) cells in control *Wfdc2*^GFP/+^ (G/+) and *Wfdc2*^GFP/LacZ^ (G/L) mice. Representative examples of Scgb1a1^high^, Scgb1a1^middle^ and Scgb1a1^low^ cells are shown in Fig. S2C. Fewer secretory cells expressed high to middle levels of Scgb1a1 in *Wfdc2*^GFP/LacZ^ mice. Data are shown as mean±s.e.m. (*n*=3 mice; ***P<0.005, ****P<0.001). *P-*values were calculated as compared with littermate controls.

To examine Wfdc2 expression at the cellular level, we tracked GFP expression in *Wfdc2*^GFP/+^ mice. This showed that a subset of basal [positive for keratin 5 (KRT5^pos^)] and club (Scgb1a1^pos^) cells express GFP (Fig. 2A,C). To adjust the *GFP* gene dosage between knock-out and heterozygous mice, *Wfdc2*^GFP/LacZ^ mice were established and used instead of *Wfdc2*^GFP/GFP^ mice. In both *Wfdc2*^GFP/+^ and *Wfdc2*^GFP/LacZ^ mice, a subpopulation of basal cells positive for KRT5 were positive for GFP (Fig. 2A,B), and GFP^pos^/KRT5^pos^ basal cells in *Wfdc2*^GFP/LacZ^ mice were fewer than those in *Wfdc2*^GFP/+^ mice (Fig. 2B).

At P1.5, Scgb1a1 expression levels in *Wfdc2*^GFP/+^ mice varied significantly, ranging from low to high (Fig. S2A). Histological analysis of *Wfdc2*^GFP/LacZ^ lungs showed far fewer Scgb1a1^high^ cells than wild type (Fig. 2C, Fig. S2B). When we graded Scgb1a1-positive cells into three categories – low, middle and high (Fig. S2C) – *Wfdc2*^GFP/LacZ^ mice contained fewer Scgb1a1^high^ and Scgb1a1^middle^ cells (Fig. 2D), suggesting that club cells in the homozygous mutants fail to terminally differentiate. Interestingly, the phenotype was only evident at the large airways of the trachea and primary bronchi, whereas, in the intralobular airways, Scgb1a1 expression was normal (Fig. S2D).

### Cilia formation is impaired in *Wfdc2*-deficient mice

**Fig. 3.**
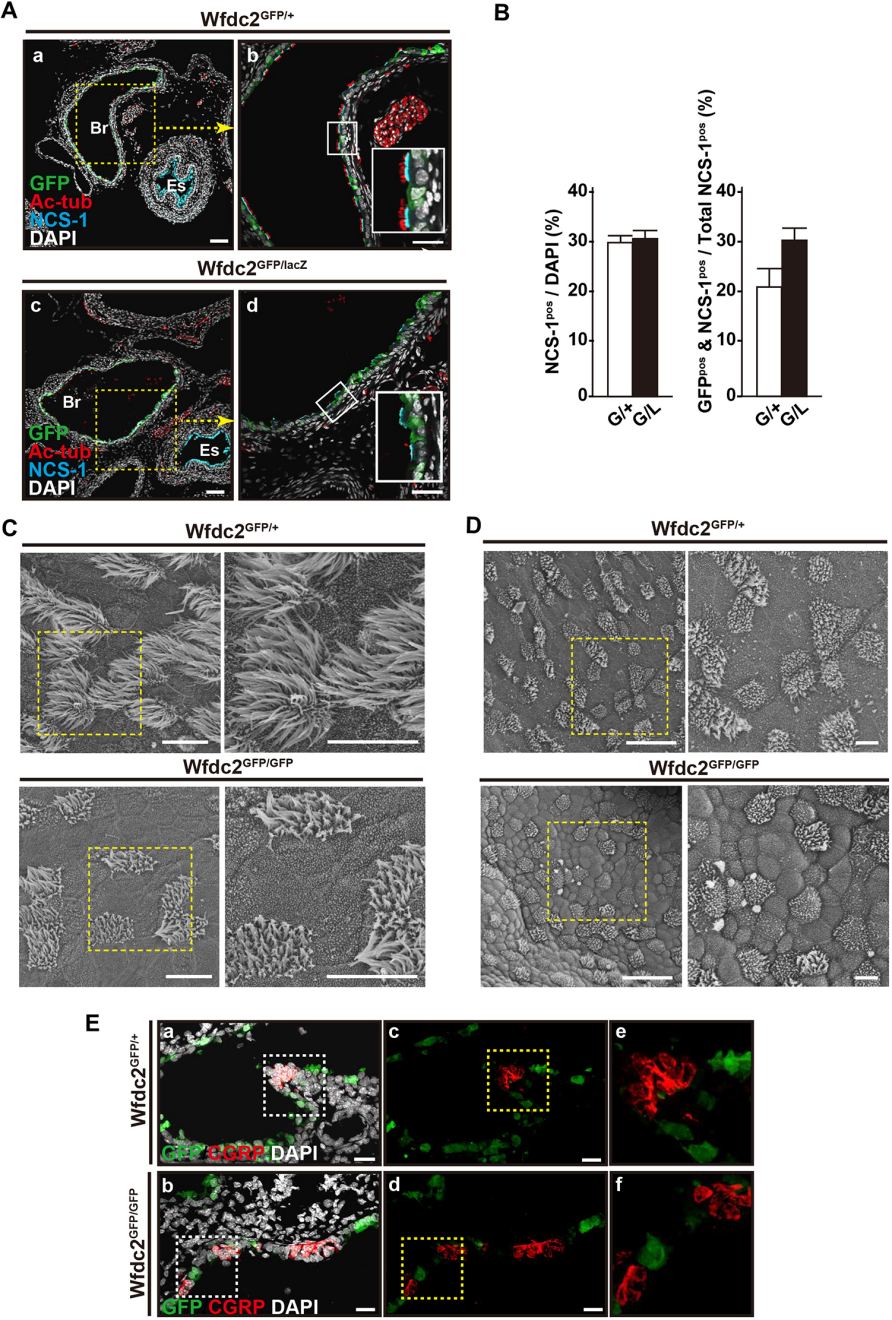
**Wfdc2 deficiency causes cilia abnormalities in proximal conducting airways.** (A) NCS-1^pos^ ciliated cells lacking Wfdc2 show poor cilia formation. Yellow dotted areas in panels a and c are enlarged in b and d, respectively. Enlargements of the white boxed areas in b and d are shown at the bottom right corner of each panel. Note that clusters of acetylated tubulin (Ac-tub)-positive cells are common in *Wfdc2*^GFP/+^ but are rarely seen in *Wfdc2*^GFP/LacZ^ mice. Green, GFP; cyan, NCS-1; red, Ac-tub; white, DAPI; Es, esophagus; Br, main bronchus. Scale bars: 50 µm. (B) The frequency of NCS-1-positive ciliated cells is similar among *Wfdc2*^GFP/+^ (G/+) and *Wfdc2*^GFP/LacZ^ (G/L) mice. (C) SEM analysis reveals shorter and less abundant cilia in the P1.5 main bronchus of *Wfdc2*^GFP/GFP^ mice. Scale bars: 10 µm. (D) SEM analysis reveals that cilia in the main bronchus of *Wfdc2*^GFP/GFP^ mice are comparable to those in *Wfdc2*^GFP/+^ mice at E18.5. Scale bars: 20 µm. (E) CGRP-positive neuroendocrine cells are largely negative for GFP. Green, GFP; red, CGRP; white, DAPI. Scale bars: 20 µm.

To explore the cause of the atelectasis, ciliated epithelial cells were visualized by staining for acetylated tubulin (Ac-tub) in combination with either Foxj1 or neuronal calcium sensor-1 (NCS-1), which is specifically expressed in ciliated cells (Fig. 3A, Fig. S3A) (Treutlein et al., 2014). The number of NCS-1^pos^ ciliated cells in the tracheobronchial area of *Wfdc2*-deficient mice was similar to control (Fig. 3B). However, the pattern of Ac-tub staining was abnormal (Fig. 3A, Fig. S3A), suggesting that the structure of the cilia was severely altered. This was confirmed by scanning electron microscopy (SEM) at P1.5 (Fig. 3C). In *Wfdc2*^GFP/GFP^ mice, the cilia were consistently shorter than in *Wfdc2*^GFP/+^ mice and their morphology was abnormal (Fig. 3C). This difference was not seen before birth (i.e. at E18.5) (Fig. 3D). Immunohistochemical (IHC) analysis showed that the clustered CGRP^pos^ neuroendocrine cells were clearly GFP negative (Fig. 3E, Fig. S3B), and the quantitative analysis showed no difference in the frequency of these cells between *Wfdc2*^GFP/+^ and *Wfdc2*^GFP/GFP^ mice (Fig. S3B).

### Abnormal lamellar body formation and surfactant processing in *Wfdc2*-deficient lungs

**Fig. 4.**
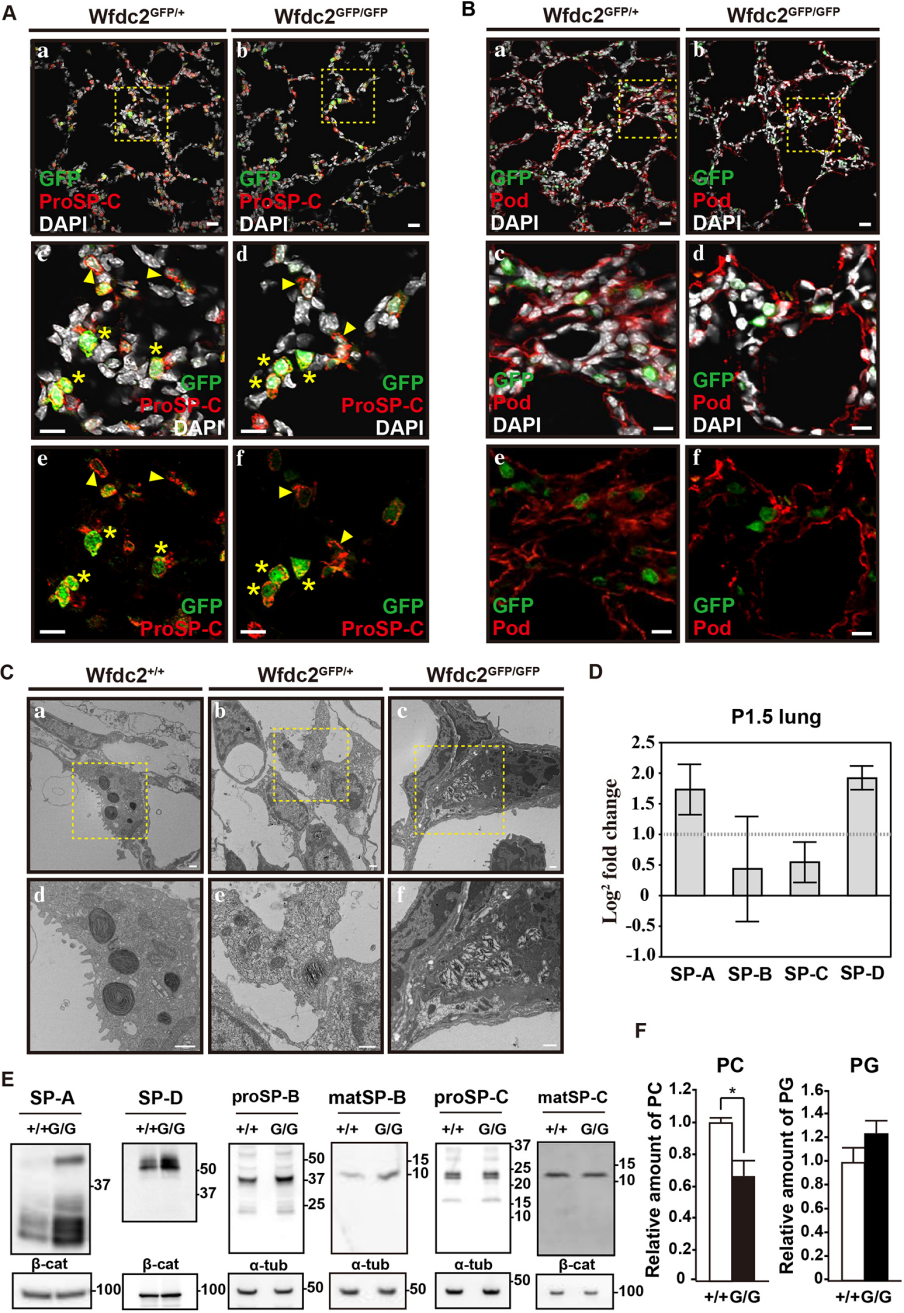
**AECIIs express proper markers but produce abnormal surfactants.** (A) P1.5 lung specimens were stained with GFP (green), ProSP-C (red) and DAPI (white); (a,c,e) *Wfdc2*^GFP/+^ mice; (b,d,f) *Wfdc2*^GFP/GFP^ mice. Yellow dotted areas in panels a and b are enlarged in c,e and d,f, respectively. Yellow asterisks indicate GFP^pos^/ProSP-C^pos^ (double-positive) cells. Yellow arrowheads indicate GFP^neg^/ProSP-C^pos^ cells. Scale bars: 30 µm. (B) P1.5 lung specimens were stained for GFP (green), podoplanin (Pod; red) and DAPI (white); (a,c,e) *Wfdc2*^GFP/+^ mice; (b,d,f) *Wfdc2*^GFP/GFP^ mice. Yellow dotted areas in panels a and b are enlarged in c,e and d,f, respectively. Scale bars: 20 µm. (C) TEM analysis of lamellar bodies in AECIIs. Yellow dotted areas in panels a-c are enlarged in d-f, respectively. Scale bars: 500 nm. (D) RNA-seq analyses of hydrosoluble surfactant proteins. mRNA expression changes of SP-A, SP-B, SP-C and SP-D are shown as log2 fold change between *Wfdc2*^+/+^ and *Wfdc2*^GFP/GFP^ lung tissues at P1.5. The dotted line at 1.0 indicates twofold upregulation in *Wfdc2*^GFP/GFP^ mice. Error bars show mean±s.e.m. (*n*=2). (E) Representative western blotting of liposoluble surfactant proteins. (Left) SP-A and SP-D are upregulated in *Wfdc2*^GFP/GFP^ mice. (Middle and right) matSP-B shows a little increase in *Wfdc2*^GFP/GFP^ mice. +/+, *Wfdc2*^+/+^ mice; G/G, *Wfdc2*^GFP/GFP^ mice; β-cat, β-catenin; α-tub, α-tubulin. (F) *Wfdc2*^GFP/GFP^ mice have low levels of phosphatidylcholine (PC) compared with *Wfdc2*^+/+^ mice, but comparable levels of phosphatidylglycerol (PG). Data are shown as mean±s.e.m. (*n*=3 mice; *P<0.05). *P*-values were determined compared to controls.

IHC analysis showed low levels of Wfdc2-GFP expression in the alveolar region of the lung (Fig. S4A) in a subfraction of cells positive for ProSP-C and ABCA3, markers of AECIIs (Fig. 4A, Fig. S4B). *Wfdc2*-deficient mice showed a higher frequency of GFP-positive AECIIs (Fig. S4B). By contrast, podoplanin-positive type I alveolar epithelial cells (AECIs) did not express GFP (Fig. 4B). To quantify GFP expression in AECIs, we used Hopx and confirmed the lack of GFP expression in Hopx-positive/ProSP-C-negative AECIs (Fig. S4D) (Yin et al., 2006; Barkauskas et al., 2013). Given that Wfdc2 is expressed in AECIIs, we speculated that the lung collapse and respiratory distress in null mutants is caused by dysfunction of these cells and that this protease inhibitor normally regulates surfactant composition through synthesis and/or recycling of surfactant components. This hypothesis is supported by the following observations. Firstly, transmission electron microscopy (TEM) of *Wfdc2*^GFP/GFP^ mice showed that mutant AECIIs were significantly impaired in the ability to form lamellar bodies at inflated regions (Fig. 4C). Secondly, RNA-seq analyses showed that mRNA levels of hydrophilic surfactant peptides (SP-A and SP-D) were significantly upregulated in *Wfdc2*^GFP/GFP^ mice (Fig. 4D). In agreement with the mRNA expression data, western blotting analyses showed increased levels of SP-A and SP-D proteins in *Wfdc2*^GFP/GFP^ mice (4.07 and 2.06 times, respectively), as compared with those of littermate controls (Fig. 4E, Table S1), and matSP-B showed a little increase (Fig. 4E, Table S1). Lastly, when the levels of two major phospholipids, PC and PG, were quantified, levels of PC but not PG were significantly lower in the lung of *Wfdc2*^GFP/GFP^ mice than in littermate controls (Fig. 4F).

### *Wfdc2*-deficient mice upregulate pro-inflammatory and defense/immune-response mRNAs

**Fig. 5.**
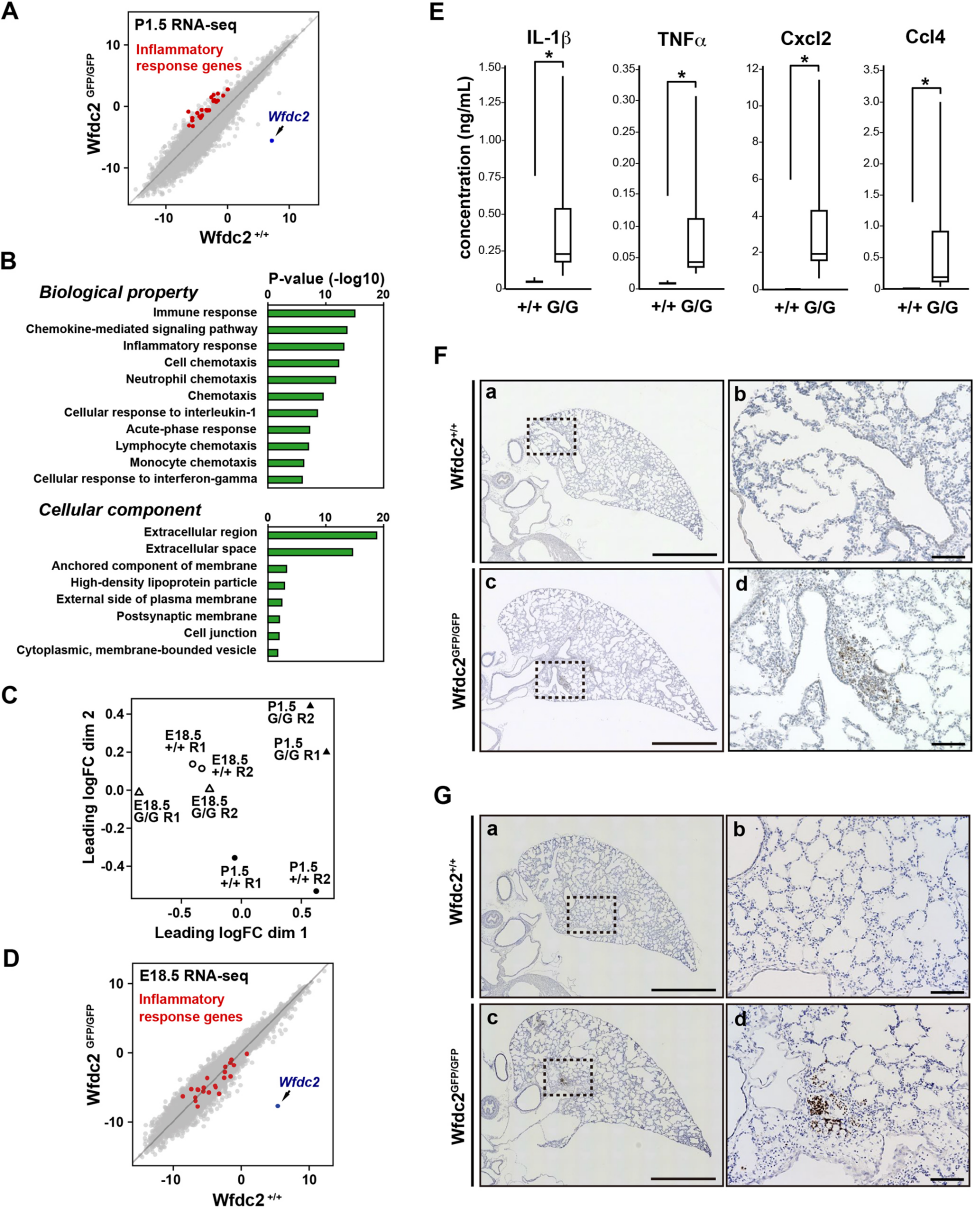
**Wfdc2 deficiency causes inflammation in the neonatal lung.** (A) RNA-seq scatter plot showing mRNA expression levels of all genes (gray dots) expressed in *Wfdc2*^+/+^ and *Wfdc2*^GFP/GFP^ lungs at P1.5. Values are indicated as log2-normalized read count per million mapped reads. *Wfdc2* mRNA is reduced, and mRNA for inflammatory response genes (red dots) is upregulated in *Wfdc2*^GFP/GFP^ mice. (B) GO term enrichment analysis of the upregulated genes (>fourfold) in *Wfdc2*^GFP/GFP^ mice. (C) Multidimensional scaling plot showing distances between RNA-seq datasets of different conditions. The plot was generated using the plotMDS function of EdgeR. White and black circles indicate *Wfdc2*^+/+^ (+/+) littermate control samples from E18.5 and P1.5, respectively; white and black triangles indicate *Wfdc2-*knockout (G/G; *Wfdc2*^GFP/GFP^) samples from E18.5 and P1.5, respectively. R, replicate #1; R2, replicate #2. (D) The gene expression profile of E18.5 *Wfdc2*^GFP/GFP^ fetuses is similar to that of *Wfdc2*^+/+^ fetuses. Only the *Wfdc2* gene expression is significantly downregulated (black arrow). Red dots indicate inflammatory response genes that are upregulated at P1.5 in *Wfdc2*^GFP/GFP^ mice. (E) Induction of acute inflammatory response proteins in *Wfdc2*^GFP/GFP^ mice at P2.5. Data are shown as mean±s.e.m. (*n*=4, *P<0.05). (F) Immunohistochemical analysis of IL-1α in neonatal lung at P1.5. Black dotted boxes are enlarged in the right panels. IL-1α-positive cells (brown) are detected in some epithelial cells in bronchioles and mesenchymal areas, and mononuclear cells appeared in alveolar spaces in *Wfdc2*^GFP/GFP^ mice. Scale bars: 1 mm (a,c); 100 µm (b,d). (G) Immunohistochemical analysis of CXCL2 in neonatal lung at P1.5. Black dotted boxes are enlarged in the right panels. CXCL2-positive cells (brown) are also detected in some epithelial cells in bronchioles and mesenchymal areas, and mononuclear cells appeared in alveolar spaces in *Wfdc2*^GFP/GFP^ mice. Scale bars: 1 mm (a,c); 100 µm (b,d).

To uncover how Wfdc2 deficiency affects lung gene expression, we used RNA-seq to profile and compare the lung transcriptome of *Wfdc2*^GFP/GFP^ and littermate control mice at P1.5. This revealed upregulation of a set of genes involved in the immune response (*P*=2.3e–17; *n*=23 genes) and its related functions, which include a number of pro-inflammatory and anti-inflammatory cytokines as well as various CC chemokines and CXC chemokines (Fig. 5A,B, Fig. S5A, Table 3). Significantly, this upregulation was not observed before birth, at E18.5 (Fig. 5C,D). Among the upregulated genes, *IL-1*α and *TNFβ* were notable, with approximately 5.4- and 4.9-fold overexpression in *Wfdc2*^GFP/GFP^ mice. The upregulation of *Ccl4*/*MIP1β* and *Cxcl2/MIP-2* was also prominent, with 11.5- and 16.0-fold overexpression in *Wfdc2*^GFP/GFP^ mice.

**Table 3.**
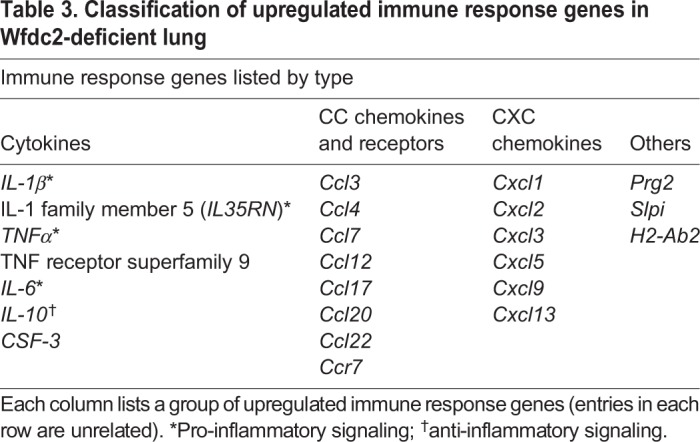
**Classification of upregulated immune response genes in Wfdc2-deficient lung**

Since inflammation is controlled at both the transcriptional and translational levels, we used ELISA to measure levels of four proteins: two cytokines (IL-1α and TNFβ) and two chemokines [CCL4/MIP1β (CC class) and CXCL2/MIP-2 (CXC class)]. Analysis of lungs from *Wfdc2*-deficient mice confirmed increases in protein products of all four molecules (Fig. 5E). Immunohistochemical analysis showed strong IL-1α and CXCL2 signals in some of the epithelial cells in the bronchus, and in mesenchymal regions of the alveolus; in addition, mononuclear cells appeared in alveolar spaces more often in *Wfdc2*^GFP/GFP^ mice than in littermate controls (Fig. 5F,G).

### Changes in protein expression and localization in *Wfdc2*-deficient mice

**Fig. 6.**
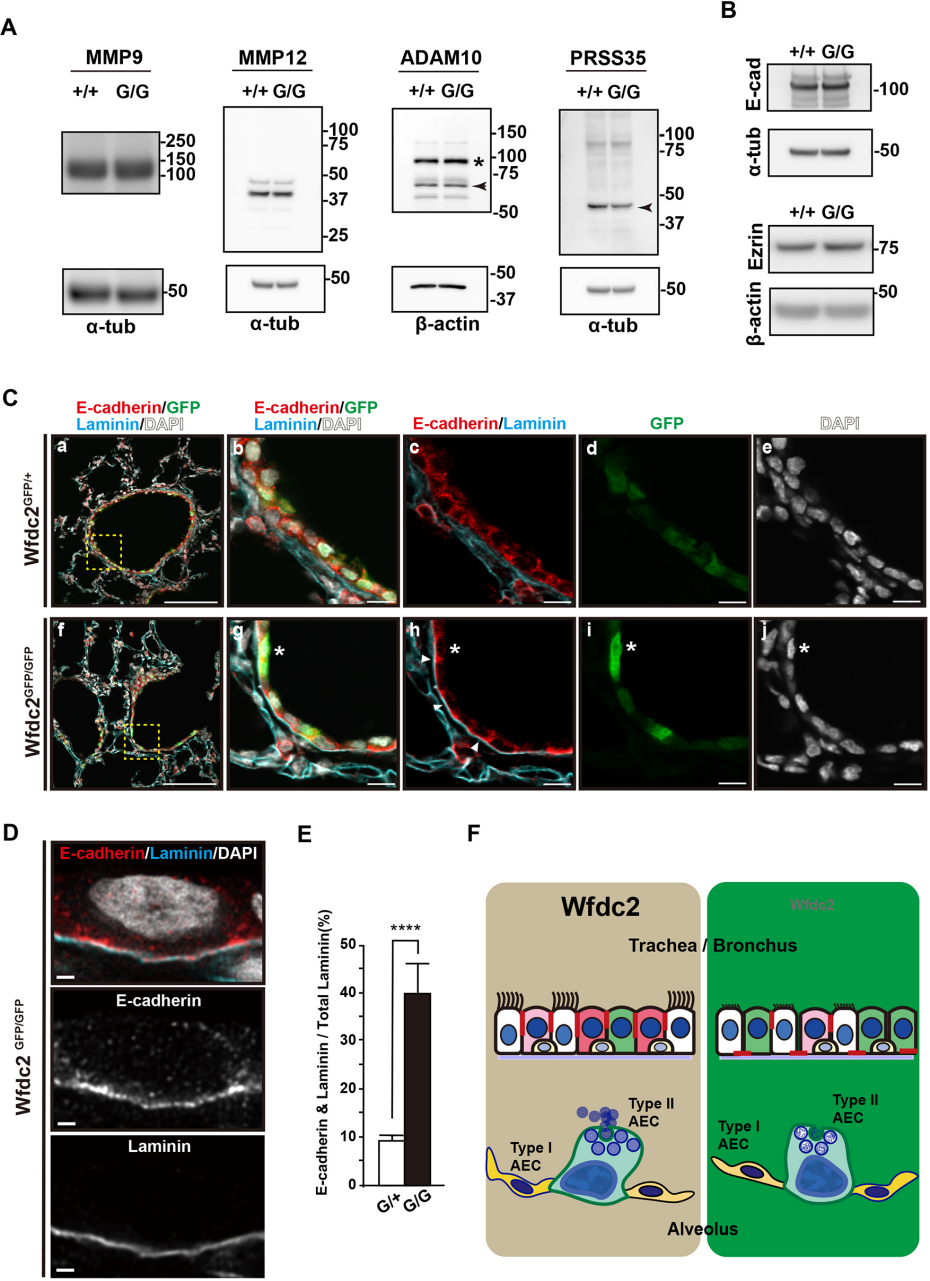
**Disturbance of homeostasis in the *Wfdc2*-deficient lung.** (A) Western blotting analysis of proteases. +/+, *Wfdc2*^+/+^ mice; G/G, *Wfdc2*^GFP/GFP^ mice. The asterisk and the arrowhead in the ADAM10 blot indicate the precursor and the active form, respectively. The arrowhead in the PRSS35 blot indicates PRSS35. (B) Western blotting analysis of E-cadherin (E-cad) and ezrin. +/+, *Wfdc2*^+/+^ mice; G/G, *Wfdc2*^GFP/GFP^ mice. (C) Mislocalization of E-cadherin in lateral walls of *Wfdc2*^GFP/GFP^ airways. Red, E-cadherin; blue, laminin; green, GFP; white, DAPI. Yellow dotted boxes in panels a and f are enlarged in b-e and g-j, respectively. E-cadherin localizes at the lateral walls of conducting airways in *Wfdc2*^GFP/+^ mice (b-e). In *Wfdc2*^GFP/GFP^ mice, E-cadherin (red in h) shows mislocalization to the basement membrane (visualized by laminin, blue in h). Note that only the *Wfdc2*^GFP/GFP^ samples show white signals at the basement membrane, as a result of overlapping E-cadherin and laminin signals (arrowheads in h). Asterisks in g-i show the cell shown in D. Scale bars: 100 µm (a,f); 10 µm (b-e,g-j). (D) Super-resolution microscopy analysis of E-cadherin and laminin signals. A high-magnification image of an epithelial cell in the conducting airway shown in C (*Wfdc2*^GFP/GFP^ mice, indicated by asterisks in Cg-Cj). The HyVolution system is used for super-resolution imaging. Single-channel images of E-cadherin and laminin are shown in the middle and the bottom panels, respectively. Scale bars: 1 µm. (E) Quantification of colocalization signals of E-cadherin and laminin at the basal side of intralobular bronchioles. Data are shown as mean±s.e.m. (*n*=3, ****P<0.001). (F) Summary of the Wfdc2-deficiency status in the lung. Wfdc2 is expressed in a subpopulation of basal cells, ciliated cells and club cells at the proximal airway, but not in neuroendocrine cells. At the distal airway, a subpopulation of AECIIs but not in AECIs express Wfdc2. Wfdc2 deficiency leads to damaged cilia, a reduction of terminally differentiated club cells, which have secretory functions (pink and red cytosol), and abnormal distribution of E-cadherin (red line). At the distal airway, proper lamellar body formation in AECIIs is also impaired. Altogether, many abnormalities arising from Wfdc2 deficiency give rise to a breakdown of the multi-layered barrier system.

In a kidney fibrosis model, Wfdc2 overexpression reportedly causes up- and down-regulation of proteases at the protein and mRNA levels, as well as at the enzymatic activity level (LeBleu et al., 2013). Therefore, the observed disruption in lung homeostasis might be caused by an imbalance of the protease/protease-inhibitor system. In the kidney fibrosis model, protein levels of Prss35 and MMP9 were upregulated in parallel with the upregulation of Wfdc2, and Wfdc2 inhibits enzymatic activity of Prss35 and MMP9 (LeBleu et al., 2013). In our lung knockout model of Wfdc2, only PRSS35 revealed a little downregulation (Fig. 6A, Table S1). We also checked two other lung-disease-related proteases, MMP12 and ADAM10. Of those, ADAM10 showed marginal changes in Wfdc2-deficient lungs at P1.5 (Fig. 6A, Table S1).

As protease levels increase, target proteins likely are digested, perturbing homeostasis and self-defense mechanisms. As a consequence, the structure of the lung tissue may not mature and may collapse. Wfdc2 target molecules might mediate cell adherence and tight junctions, or be a structural part of the alveolar epithelial barrier, an idea supported by recent studies in the mouse colon (Parikh et al., 2019). To track the integrity of intercellular junctions, expression levels of the adherence-junction protein E-cadherin and the tight-junction protein ezrin were measured and their localization was analyzed (Fig. 6B-D, Table S1). Although the expression levels of E-cadherin and ezrin were not different among the mice (Fig. 6B, Table S1), in *Wfdc2*-deficient mice, E-cadherin was often expressed in an unusual pattern, at the basal side of the epithelial cells of the intralobular conducting airway, compared with at the lateral wall in wild-type controls (Fig. 6C,D).

## DISCUSSION

Our study in the lung has presented, for the first time, evidence that the protease inhibitor Wfdc2 plays an important role in a wide range of aspects of the barrier mechanisms that protect respiratory airway epithelium from damage, which include lamellar body formation in AECIIs and mucociliary clearance machinery in bronchi (Fig. 6E). We have shown here that the mice develop atelectasis as a consequence of Wfdc2 deficiency, leading to neonatal death.

Ninety percent of pulmonary natural surfactant is composed of lipids, and PC makes up approximately 80% of the lipid. PC functions to reduce the surface tension, which prevents collapse of alveoli (Echaide et al., 2017; Parra and Pérez-Gil, 2015). Our study has shown that PC levels significantly decreased in response to Wfdc2 deficiency, likely due to an impairment of synthesis and/or recycling of PC (Chakraborty and Kotecha, 2013). Given that the reduction of PC concentration increases the surface tension (Hallman et al., 1991), a decrease in PC may be linked to progressive atelectasis and respiratory failure in *Wfdc2*-deficient mice. This is also indirectly supported by clinical reports showing that patients of acute respiratory distress syndrome (ARDS) accompanied by severe atelectasis exhibit decreased levels of PC (Cogo et al., 2007; Dushianthan et al., 2014; Gregory et al., 1991). Meanwhile, we also found that SP-A and SP-D levels were increased in *Wfdc2*-deficient mice. Given that an increase of the SP-A/PC ratio experimentally causes an increase of surface tension (Hallman et al., 1991), the upregulation of SP-A and the downregulation of PC in *Wfdc2*-deficient mice may accelerate lung atelectasis. Abnormal processing of surfactants may also cause secondary changes in lung physiology at birth: mice lacking other surfactant components – SP-B and ABCA3 – have been shown to die of RDS (Clark et al., 1995; Cheong et al., 2007; Fitzgerald et al., 2007; Hammel et al., 2007).

Club cells produce SP-A, SP-B and SP-D as well as an inhibitor of phospholipase A2 (CC10) (Han and Mallampalli, 2015), and have crucial secretory functions contributing to the mucus pool. The mucus, together with surfactants, interacts with and subsequently kills pathogens to prevent their dissemination. The mucus is then cleared by a constant upward flow generated by cilia. Therefore, the decreased number of mature club cells in *Wfdc2*-deficient mice may lead to a breakdown of the self-defense barrier function. Even worse, the impairment of cilia formation in *Wfdc2*-deficient mice may fail to clear pathogens in the mucus.

Various types of lung disorders, such as the chronic obstructive lung disease, are known to be associated with protease/antiprotease imbalance (Abboud, 2012; Goopts et al., 2009). At present, the target proteases of Wfdc2 are unclear. Until now, multiple examples carrying dysfunctions in other protease systems have been presented to be associated with lung diseases: neutrophil elastase/α1-antitrypsin imbalance causing human emphysema (Goopts et al., 2009; Meyer and Jaspers, 2015); mouse α1-antitrypsin (*serpina1*) knockout leading to spontaneous emphysema (Borel et al., 2018); and chymotrypsin-like elastase 1 responsible for emphysema, proven by an antisense oligo model for the α1-antitrypsin deficiency (Joshi et al., 2018). However, the phenotype of the Wfdc2-deficient mice is different from those of the above-described mice. Moreover, although many Wfdc family genes are closely located on the same chromosome, possibly as a consequence of rapid evolutionary changes (Hurle et al., 2007), our single-knockout model indicates that Wfdc2 exhibits non-redundant yet diverse functions in the lung.

During the preparation of the manuscript, a new report was published showing that human WFDC2 is downregulated in goblet cells in inflammatory bowel disease (IBD) patients and participates in the pathogenesis of IBD (Parikh et al., 2019). Analysis in the colon has shown that WFDC2 has bactericidal activity, preserves the integrity of tight junctions and participates in forming a mucus layer to block bacteria penetration into epithelial cells. This finding raises another possibility: that mislocalization of the adherens-junction molecule E-cadherin in *Wfdc2*-deficient lung epithelia may reflect the loss of epithelial barrier integrity. Indeed, some major proteases, such as ADAM15, kallikrein 6 and MMPs, reportedly mediate E-cadherin shedding and affect cell-cell adhesion (Biswas et al., 2010; Klucky et al., 2007; Kwon et al., 2014; Najy et al., 2008; Symowicz et al., 2007; Zuo et al., 2011). Since the protein levels of the proposed Wfdc2-target proteases in the kidney fibrosis model did not change significantly in our knockout lung study, the target proteases of Wfdc2 may be tissue specific. Therefore, future studies in the lung system will address which proteases are targeted by Wfdc2 and whether the effect of their interactions extends to epithelial integrity in multiple tissues.

Collectively, our results suggest that Wfdc2 deficiency causes impairments in a series of barrier mechanisms required to protect respiratory airway epithelium. The deficiency not only induces disruption of surfactants, which leads to a collapsed lung, but also causes insufficient mucociliary clearance due to damaged cilia, which impedes a constant upward flow of respiratory secretion to the mouth (Fig. 6E). In the lung, since Wfdc2 is induced after birth and plays crucial roles in preventing atelectasis and possibly maintaining the barrier function, the decrease in WFDC2 expression might be a factor in human respiratory failure, an issue that should be addressed in future studies.

## MATERIALS AND METHODS

### Mouse ES cell culture and generation of GFP knock-in mice

Mouse TT2 embryonic stem cells (ES cells) were used to generate GFP knock-in mice. ES cells were cultured on 0.1% gelatin-coated plates in high-glucose DMEM, L-glutamine, sodium pyruvate (Thermo Fisher Scientific) containing 15% FBS (Hyclone, Thermo Fisher Scientific), 0.1% non-essential amino acids, 50 U/ml penicillin (Sigma-Aldrich) and streptomycin (Nacalai Tesque), 0.1 mM 2-mercaptoethanol (Millipore) and 1000 U/ml LIF supplement (ESGRO, Millipore). IRES-EGFP-rabbit β-globin-polyA cDNA was integrated into a pKSTKNeoLoxP vector carrying PGK-neo-polyA for positive selection and the HSV-tk gene for negative selection (Fig. S1A) (Nagamatsu et al., 2006). The targeting vector contains a 6.3-kb long arm, carrying a part of exon 1, and a 2 kb short arm, encompassing exon 3 and intron 3, from 129-derived genomic DNA. The vector lacks a part of exon 1 and the entire exon 2 (Fig. S1A). A targeted vector carrying *EGFP* cDNA was linearized with *Not*I and electroporated into TT2 ES cells, which were then selected in 250 mg/ml (active weight) neomycin (G418; Invitrogen) for 10 days and ganciclovir for 7 days (Sigma-Aldrich). After confirming recombination of neomycin-resistant colonies, the ES cells were aggregated with ICR mouse morulae to generate chimeric mice. After confirming germline transmission from chimeric mice, heterozygous mice were crossed with CAG-Cre mice to delete the drug-resistant marker, PGK-neo-polyA (*Wfdc2*^GFP/+^), and then homozygous mice (*Wfdc2*^GFP/GFP^) were generated. Southern blotting and genomic PCR analyses verified that the *Wfdc2* gene was deleted (Fig. S1B and S1C); RNA-seq did not detect any *Wfdc2* mRNA in the mutant mouse lung (Fig. S1D). A second mutant line, with the *lacZ* gene knocked in at the *Wfdc2* locus, was generated and intercrossed with *Wfdc2-*knockout mice, generating mutant mice carrying both *GFP* and *lacZ* (*Wfdc2*^GFP/lacZ^ mice) (Fig. S1A).

### Mice

Mouse husbandry and experiments were carried out under the guidelines of Yokohama City University, Japan, and all animal experiments were approved by the Committee for Animal Care and Use at Yokohama City University. *CAG-Cre* mice were reported previously (Glaser et al., 2009). The *lacZ* knock-in mice were generated from ES cells obtained from EUCOMM.

### Southern blotting

Ten µg of genomic DNA was prepared from E18.5 fetuses, as described previously with slight modifications (Ohbo et al., 1996), and digested with the indicated restriction enzymes (Fig. S1B), separated by electrophoresis on 0.7% agarose gels in TBE buffer, transferred to Hybond-N (RPN303B, Amersham) and hybridized with the probes indicated in Fig. S1A.

### Genomic PCR

Primer sequences for genotyping PCR are listed in Table S2.

### Antibodies

Antibodies for IHC analysis are listed in Table S3.

### Histology and immunostaining

Fixation and immunostaining of lung were done as described previously (Shirakawa et al., 2013). Briefly, neonatal lung was fixed by perfusion with 4% paraformaldehyde (PFA) for 4 h (P0.5 and older). Embryonic and fetus lung was immersed in 4% PFA for 1 h (E11.5) and 4 h (E14.5, E18.5), respectively. After mounting in O.C.T. compound (Tissue-Tek), blocks were sliced and subjected to staining as follows. The sections were initially incubated for 30 min with PBS supplemented with 2% BSA (BSA/PBS), and then incubated for either 2 h at room temperature or overnight at 4°C with an appropriate primary antibody, followed by incubation with a secondary antibody for 1 h at room temperature (Table S3). The sections were mounted with ProLong Gold (Thermo Fisher Scientific) and observed by confocal laser microscopy (FV-1000; Olympus). For hematoxylin and eosin (H&E) staining, lung and heart were embedded in paraffin, sectioned and stained with H&E. For 3,3′-diaminobenzidine (DAB) staining, the sectioned specimens were incubated with a primary antibody overnight at 4°C, and then specimens were incubated with a secondary antibody conjugated with horseradish peroxidase (HRP) and reacted with 0.05% DAB and 0.015% hydrogen peroxide for 8 min at room temperature. *Wfdc2*^GFP/+^ and *Wfdc2*^GFP/LacZ^ mice were used for all the quantification experiments for the frequency of GFP-positive cells in epithelial cells. The measurements of the GFP frequency in neuroendocrine cells, AECIs and AECIIs, as well as the measurements of the overlapped region between E-cadherin and laminin, were performed blinded. The measurements of the GFP frequency in the basal cells, the ciliated cells and the club cells were performed non-blinded. Super-resolution fluorescence signals were acquired with a microscope system Leica TCS SP8 (Leica Biosystems) equipped with HyD detectors and a hybrid super-resolution (HyVolution) system. *Z*-section images were captured using the HyD detectors, and the raw data were deconvolved using Huygens (Scientific Volume Imaging) in the HyVolution package with default parameters.

### Transmission and scanning electron microscopy

Mice were fixed by perfusion using 0.1 M phosphate buffer (pH 7.4) containing 2% glutaraldehyde and 2% paraformaldehyde in 0.1 M phosphate buffer (pH 7.4). Fixed specimens were cut into small blocks and immersed in the same fixative at room temperature for 2 h. Subsequently, after washing with 0.1 M phosphate buffer, the blocks were post-fixed with 1% OsO_4_ at 4°C for 1 h. After washing with distilled water, the blocks were stained with 4% solution of uranyl acetate at room temperature for 1 h, then dehydrated in graded concentrations of ethanol solutions. For TEM observation, the lung blocks were replaced by propylene oxide, embedded in Epon812 resin (TAAB Laboratories Equipment) and polymerized at 60°C for 48 h. The ultra-thin sections of lung were made using Reichert Ultracut N Ultramicrotome (Leica Microsystems) and stained with 2% uranyl acetate in 70% ethanol and 0.4% lead citrate. The sections were analyzed by an H-7500 transmission electron microscope (Hitachi) operated at 80 kV. For SEM observation, the dehydrated blocks of trachea and bronchus were put in t-butyl alcohol, and then freeze-dried with VFD-21S (vacuum device). After the dried blocks were coated with platinum-palladium using an E102 ion coater (Hitachi), we imaged the lumen of the trachea and bronchus with an S-4800 scanning electron microscope (Hitachi) operated at 10 kV.

### RNA purification, cDNA construction and qRT-PCR

Total RNA was extracted from lung using Isogen (Nippon Gene) according to the manufacturer's instructions and treated with RQ1 DNase (Promega) at 37°C for 30 min. The total RNA was used for first-strand cDNA synthesis with Superscript III (Life Technologies). Expression levels were determined by Applied Biosystems 7900HT Fast Real Time PCR System using FastStart SYBR Green Master (Roche). Primers specific for each gene are provided in Table S2.

### Western blotting

Western blotting was performed as described (Shirakawa et al., 2013). Briefly, whole-cell extracts from lung were resolved by sodium-dodecyl-sulfate PAGE (SDS-PAGE) with a 7.5% (SP-D and ADAM10), 12.5% (SP-A), 10-20% (proSP-B, matSP-B, proSP-C, matSP-C, MMP9) gradient gel (ePAGEL, E-R1020L, Atto), and 5-20% (MMP12, PRSS35, E-cadherin, ezrin) gradient gel (ePAGEL, E-R520L, Atto). The proteins were then transferred to polyvinylidene difluoride (PVDF) membranes (Immobilon-P; Merck Millipore). Membranes were incubated with the indicated antibodies overnight at 4°C, followed by incubation with an appropriate HRP-conjugated secondary antibody for 1 h at room temperature (Table S3). Positive signals were detected and visualized with Chemi-Lumi One L or Super reagents (Nacalai Tesque). Antibodies for western blotting analysis are listed in Table S3. The band intensities were quantified using the LAS3000 mini system and Multi-Gauge version 2.3 (Fuji Film). The results of western blot quantification are summarized in Table S1.

### RNA-seq

Total lung RNA was purified using Isogen (Nippon Gene) according to the manufacturer's instructions. Quality control assessment of RNA was done using Bioanalyzer (Agilent) with RNA 6000 Nano Kit (Agilent). Genomic DNA was digested using RQ1 DNase (Promega) at 37°C for 30 min, and the resulting RNA was used for library preparation using NEBNext Ultra Directional RNA Library Prep Kit for Illumina (NEB). The libraries were sequenced using either Illumina HiSeq2500 or GAIIx*.*

### Data analysis

RNA-seq reads were quality- and adapter-trimmed with Trim Galore (version 0.4.0) (http://www.bioinformatics.babraham.ac.uk/projects/trim_galore/) after 3′ ends of reads longer than 36 nucleotides were removed using an NGS QC Toolkit (version 2.3.1). The trimmed reads were mapped onto mm10 using TopHat (version 2.1.1) (Trapnell et al., 2009) with default parameters with a guide by a gtf file containing reference mRNA coordinates. Total number of sequencing reads and mapping efficiency are shown in Table S4. Read counts and quantification were done using SeqMonk (version 1.44.0) (http://www.bioinformatics.babraham.ac.uk/projects/seqmonk/) and the values were normalized as log2 reads per kilobase per million mapped reads (RPKM) for downstream analyses. Gene Ontology (GO) analysis was performed using DAVID (version 6.8) using all mouse genes as background (Huang et al., 2009).

### ELISA and colorimetric/fluorometric assay

ELISA was performed as per the manufacturer’s protocol (Quantikine^®^ ELISA; R&D systems). Briefly, lung tissues were homogenized with Cell Lysis Buffer 2 provided by R&D Systems. Proteins of standard, control and samples were incubated with monoclonal antibodies specific for IL-1α, TNFβ, CCL4 and CX2 individually pre-coated onto microplates for 2 h at room temperature. After washing five times, HRP-conjugated antibodies against individual antigens were incubated for 2 h at room temperature. After again washing five times, a substrate solution was added to the wells. After the developing reaction, the stop solution was added and the intensity of color was measured by Sunrise™ (Tecan Life Sciences). A PG/cardiolipin assay kit (MET-5024, CELL BIOLABS) and PC assay kit (ab83377, abcam) were used to measure PG and PC, respectively. To measure PG, lung tissues were homogenized and glycerol in the tissues was extracted by MeOH/chloroform/1 M NaCl and subsequently treated with lipase to hydrolyze PG. The yielded glycerol was then phosphorylated and oxidized to produce hydrogen peroxide, which subsequently reacted with the fluorometric probe. To measure PC after homogenization of lung specimens, the extracts were incubated with the OxiRed Probe supplemented with hydrolysis enzyme. The intensity of color was measured by Wallac 1420 ARVOMX (Perkin Elmer).

### Statistical analyses

Statistical data are presented as means±standard error of mean (s.e.m.). Significance is indicated with asterisk(s): *P<0.05, **P<0.01, ***P<0.005, ****P<0.001.

## Supplementary Material

Supplementary information
