# Supplementary material for: Lack of whey acidic protein (WAP) four-disulfide core domain protease inhibitor 2 (WFDC2) causes neonatal death from respiratory failure in mice
Source: Dis Model Mech. 2019 Nov 12;12(11):dmm040139. doi: 10.1242/dmm.040139 (PMC6899016; doi:10.1242/dmm.040139)
Supplement: Supplementary information [file dmm-12-040139-s1.pdf]

# Fig. S1

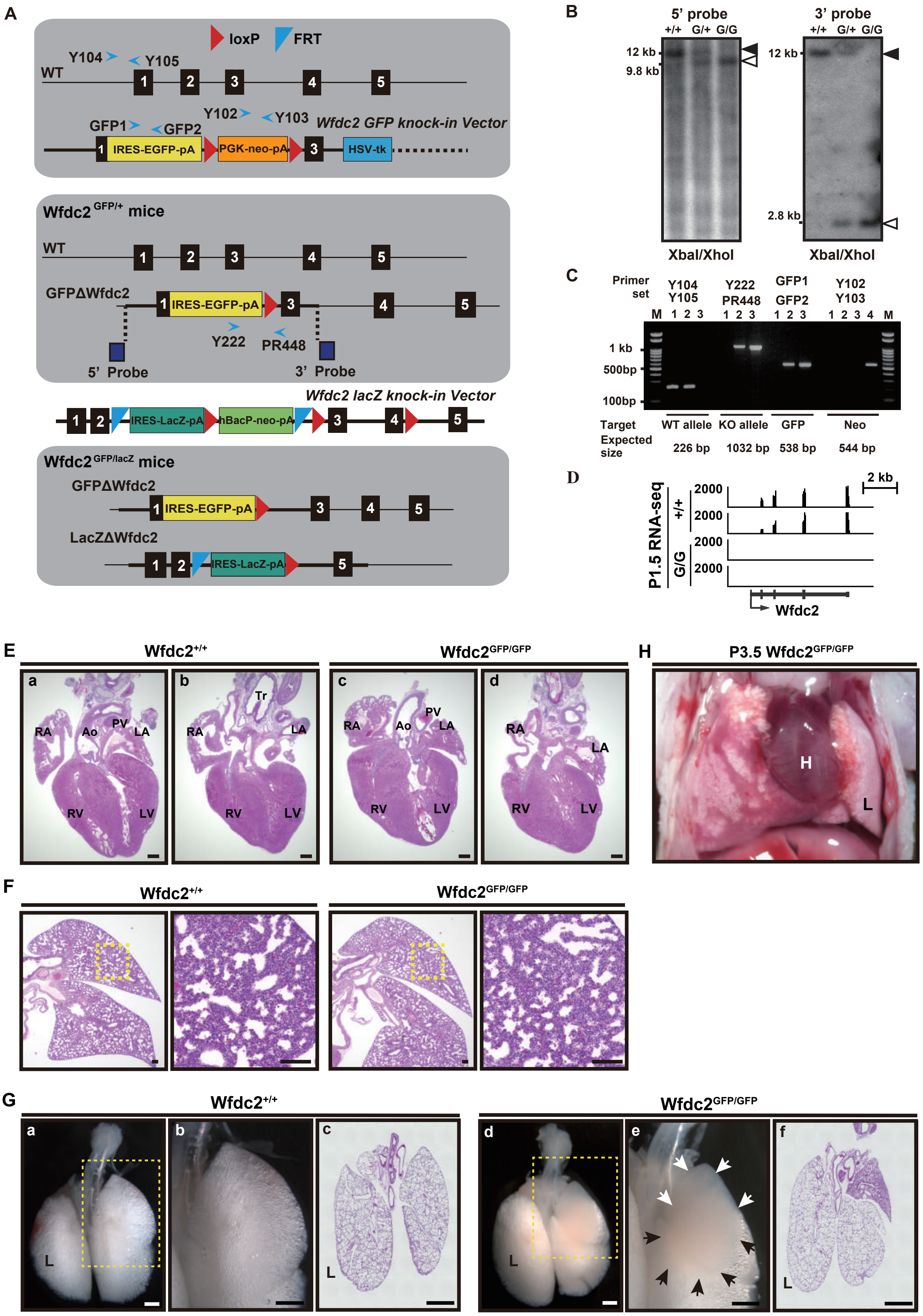


Disease Models & Mechanisms • Supplementary information

**Fig. S1.**

**Gene knockout strategy, confirmation of proper recombination and abolishment of Wfdc2 expression, and anatomical analyses of heart and embryonic lung**

**(A)** Schematic diagram of our gene knock-out strategy. GFP knock-in allele was generated by inserting the GFP knock-in vector carrying IRES-GFP-pA, PGK-neo-pA and HSV-tk-pA by homologous recombination in mouse TT2 ES cells. After deletion of the drug resistance gene, PGK-neo-pA, by crossing CAG-Cre mice, heterozygous (Wfdc2$^{GFP/+}$) mice were intercrossed to generate homozygous (Wfdc2$^{GFP/GFP}$) mice. To adjust the GFP copy number, we also generated lacZ insertion (Wfdc2$^{lacZ/+}$) mice and crossed them with Wfdc2$^{GFP/+}$ mice, to generate Wfdc2$^{GFP/lacZ}$ mice.

**(B)** Southern blotting analysis of E18.5 fetus. Two flanking probes, a 5'-probe and a 3'-probe, were used to confirm proper recombination. Location of the probes is indicated in Fig. S1A (Middle gray area). Closed arrowheads indicate wildtype alleles. Open arrowheads indicate recombined alleles. +/+; Wfdc2$^{+/+}$, G/+; Wfdc2$^{GFP/+}$, G/G; Wfdc2$^{GFP/GFP}$.

**(C)** PCR confirmation that the *Wfdc2* gene is knocked out of the genome. The location of primers is shown in Fig. S1A (Upper and middle gray areas). Genomic DNA from wild type (lane 1, Wfdc2$^{+/+}$), heterozygous (lane 2, Wfdc2$^{GFP/+}$) and homozygous (lane 3, Wfdc2$^{GFP/GFP}$) mice after the deletion of PGK-neo-polyA were used for genotyping PCR. Genomic DNA from heterozygous mice before the deletion of PGK-neo-polyA was used for positive control of neomycin gene PCR (lane 4). M; Molecular weight marker. Expected sizes of amplicons is indicated.

**(D)** Wfdc2 mRNA expression was abrogated in P1.5 Wfdc2$^{GFP/GFP}$ mice. +/+; Wfdc2$^{+/+}$, G/G; Wfdc2$^{GFP/GFP}$. Snapshots from two independent RNA-sequencing analyses are shown.

**(E)** Frontal sections of hearts at P1.5 show no anomalies in Wfdc2$^{GFP/GFP}$ mice. (a, c); Wfdc2$^{+/+}$ mice. (b, d); Wfdc2$^{GFP/GFP}$ mice. RA: right atrium, RV: right ventricle, LA: left atrium, LV: left ventricle, Ao: aorta, PV: pulmonary vein, Tr: trachea. The scale bar represents 2 mm.

**(F)** HE staining of E18.5 lung specimens. Yellow dotted boxes are enlarged in right panel. Lung structure defined by HE staining is indistinguishable between Wfdc2$^{+/+}$ and Wfdc2$^{GFP/GFP}$ fetuses. The scale bar represents 100 μm.

**(G)** Dorsal views of fixed, lung specimens. Boxed areas in (a) and (d) are enlarged in (b) and (e), respectively. Both black and white arrows in (e) indicate atelectasis. (c, f); HE staining of section of (a) and (d), respectively. Note that the upper right lobe in (f) shows atelectasis. L; left lobe. The scale bar represents 1 mm.

**(H)** Progressive lung atelectatic areas in Wfdc2$^{GFP/GFP}$ mice at P3.5. L; left lobe. H: heart.

Fig. S2

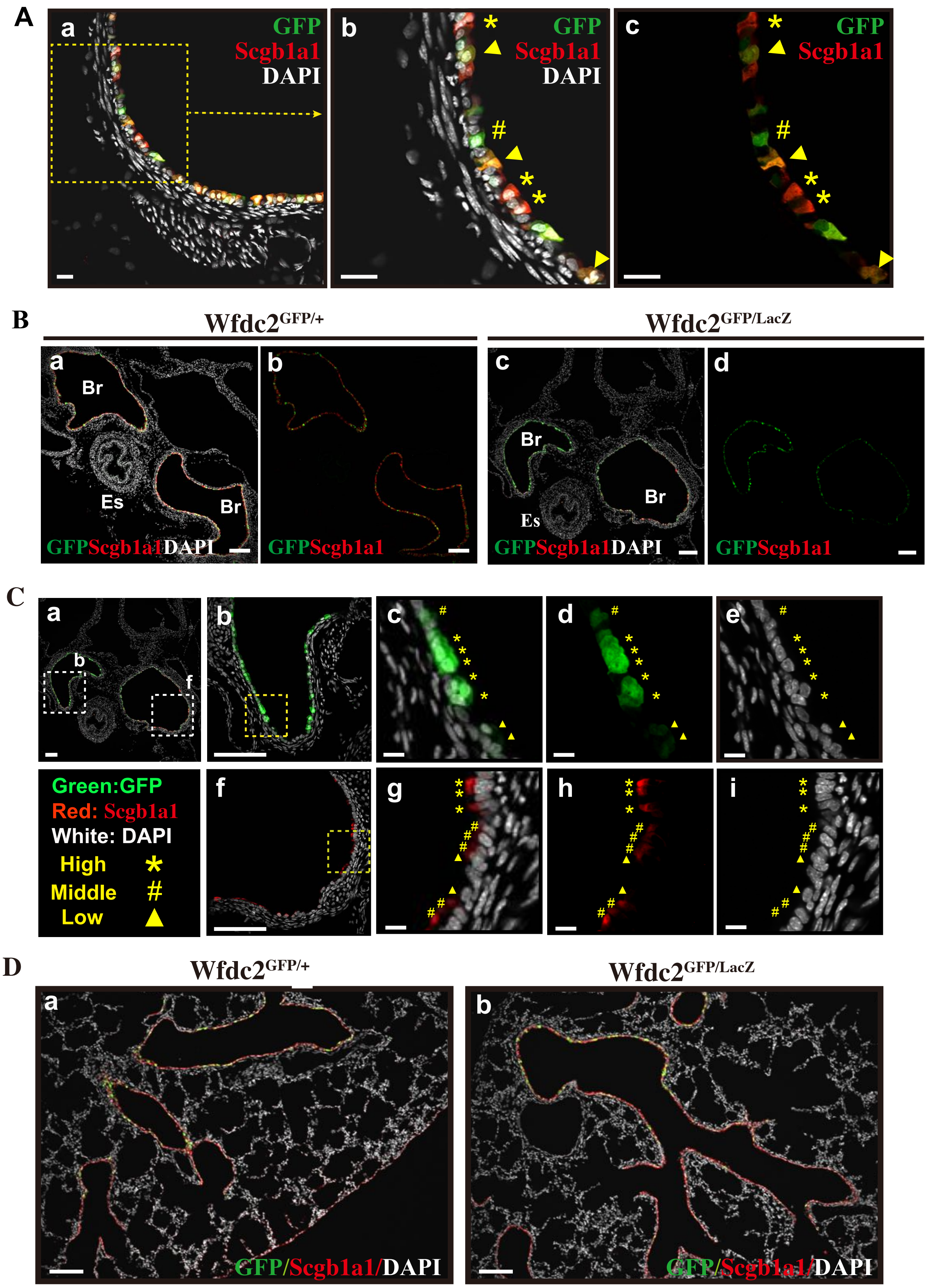


Disease Models & Mechanisms • Supplementary information

**Fig. S2. Histological characterization of Scgb1a1-positive epithelial cells in neonatal lung**

**(A)** Representative example of Wfdc2$^{GFP/+}$ bronchus. GFP-positive cells show a variety of Scgb1a1 expression levels in the main bronchus. Cells labeled with # mark indicate GFP-only cells. Arrowheads indicate cells double-positive for Scgb1a1 and GFP. Asterisks indicates cells only expressing Scgb1a1. The scale bar represents 20 µm.

**(B)** A representative example showing the bronchus of the Wfdc2$^{GFP/GFP}$ mouse which had fewer Scgb1a1$^{pos}$ cells than Wfdc2 $^{GFP/+}$ mice. The scale bar represents 100 µm.

**(C)** High (*), middle (#) and low (arrowhead) expression for GFP (c, d, e) and Scgb1a1 (g, h, i) are shown as a representative example of GFP/Scgb1a1 signal strength. Left and right dotted areas in (a) are enlarged in (b) and (f). Yellow dotted areas in (b) and (f) are enlarged in (c, d, e) and (g, h, i), respectively. The scale bar represents 100 µm in (a b, f) and 10 µm in (c, d, e, g, h, i).

**(D)** Scgb1a1$^{pos}$ cells are not altered at intralobular small airways. GFP in green, Scgb1a1 in red and DAPI in white. The scale bar represents 100 µm.

## Fig. S3

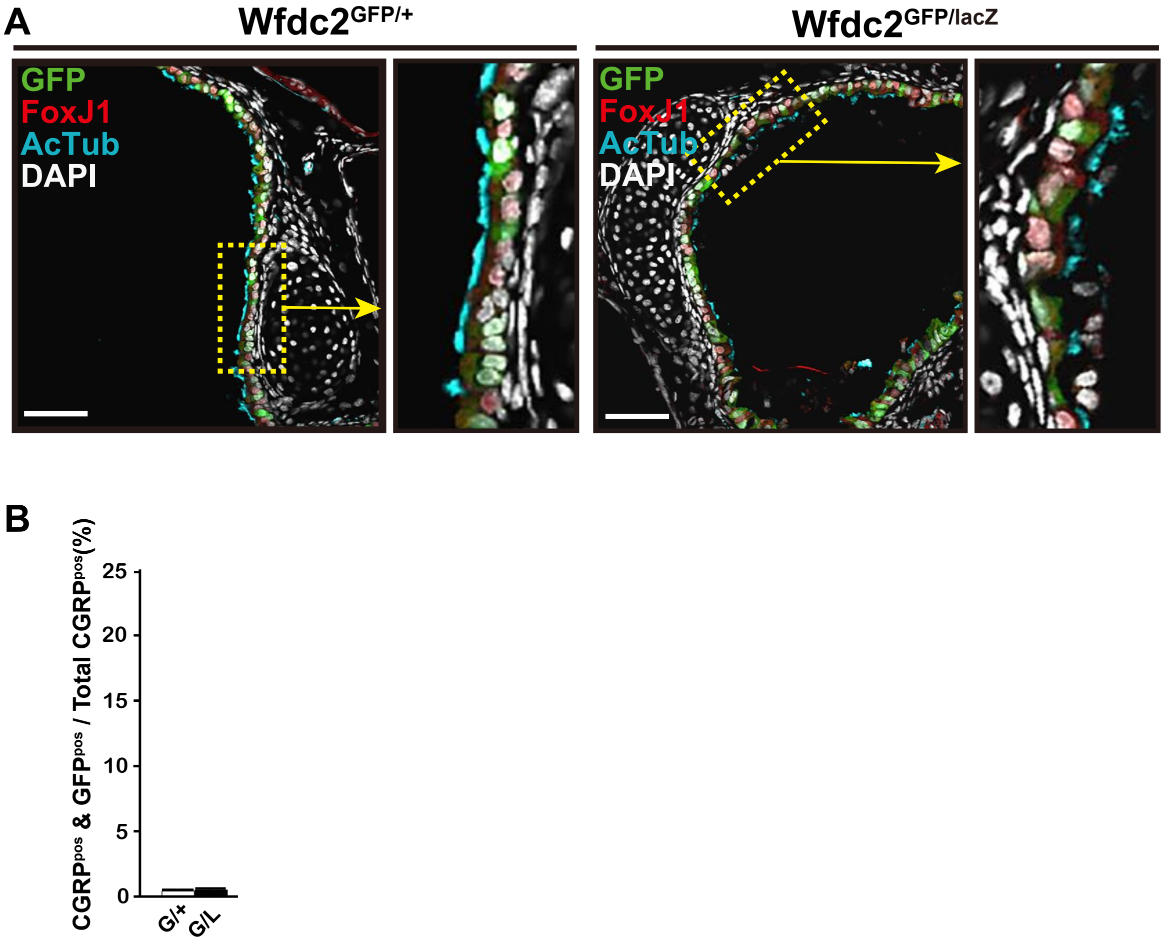


**Fig. S3 Abnormal cilia formation in Wfdc2 deficient mice**

(A) Foxj1-positive cells lacking Wfdc2 show poor cilia formation. Cyan; Ac-tubulin, Green; GFP, Red; Foxj1, White; DAPI. Yellow dotted boxes are enlarged in the right panel, respectively. The scale bar represents 50 µm.

**(B)** Quantification of $GFP^{pos}/CGRP^{pos}$ (double positive) cells among total $CGRP^{pos}$ cells in littermates of $Wfdc2^{GFP/+}$ and $Wfdc2^{GFP/LacZ}$.

Disease Models & Mechanisms • Supplementary information

Fig. S4

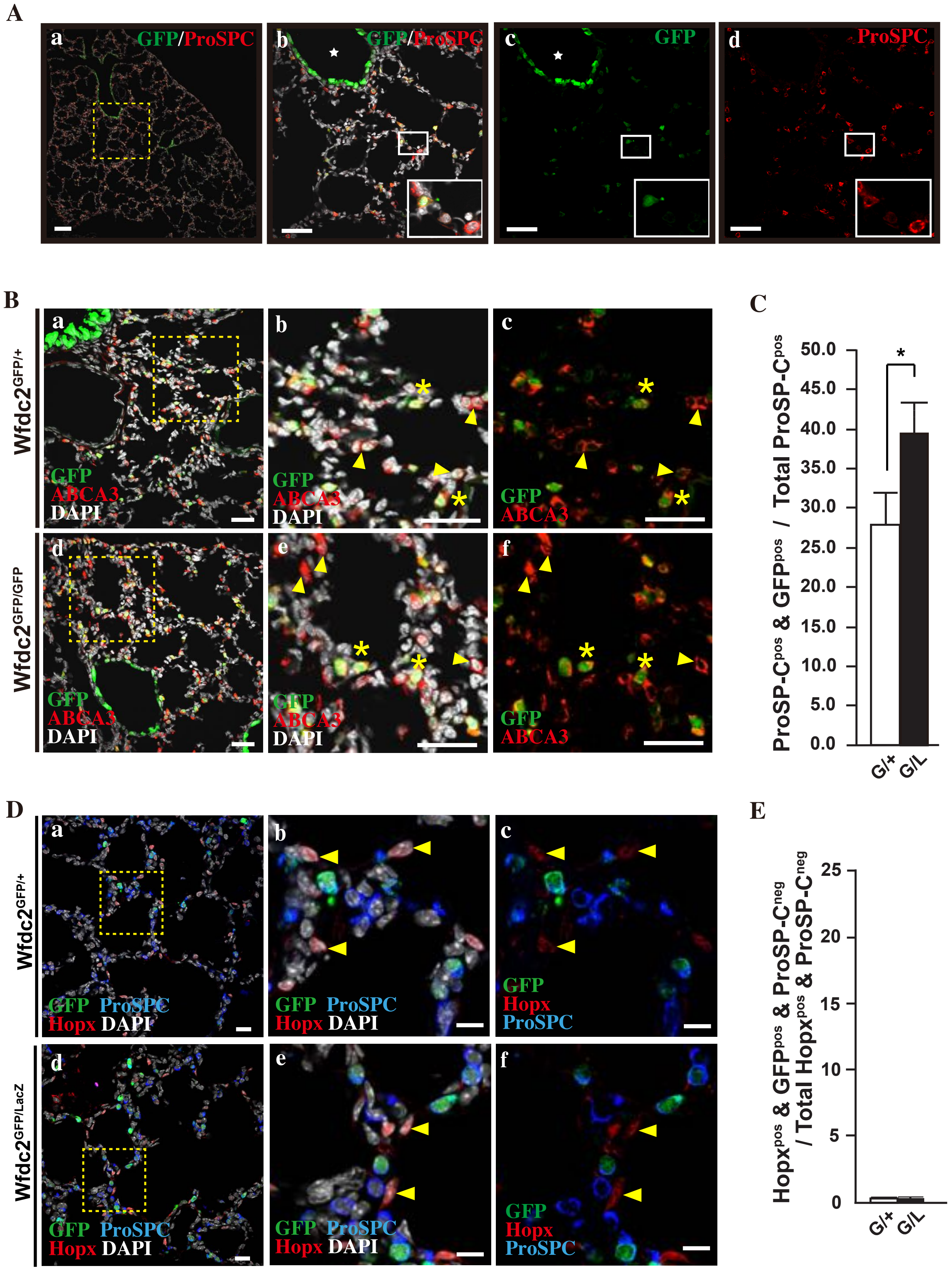


Disease Models & Mechanisms • Supplementary information

**Fig. S4. AECII-marker expression is not affected in Wfdc2 deficient mice**

**(A)** GFP signal strength at AECIIs is weaker than that at bronchioles (star). Yellow dotted areas in (a) is enlarged in (b), (c) and (d). Enlargement of the white boxed areas are shown in the bottom right-hand corner of each panel (b, c and d). The scale bars represent 100 µm in (a) and 50 µm in (b, c and d).

**(B)** P1.5 lung specimens were stained with GFP (green), ABCA3 (red) and DAPI (white). (a, b, c);Wfdc2$^{GFP/+}$ mice. (d, e, f);Wfdc2$^{GFP/GFP}$ mice. Yellow dotted areas in (a) and (d) are enlarged in (b, c) and (e, f), respectively. Yellow asterisks indicate GFP$^{pos}$/ABCA3$^{pos}$ (double positive) cells. Yellow arrowheads indicate GFP$^{neg}$/ABCA3$^{pos}$ cells. The scale bar represents 30 µm.

**(C)** Quantification of GFP$^{pos}$/ProSP-C$^{pos}$ cells among total ProSP-C$^{pos}$ cells in littermates of Wfdc2$^{GFP/+}$ and Wfdc2$^{GFP/LacZ}$. Data are shown as mean ±SEM (n=3 mice; *$p<0.05$). p values were determined compared to controls.

**(D)** P1.5 lung specimens were stained with GFP (green), Hopx (red), ProSP-C (blue) and DAPI (white). (a, b, c);Wfdc2$^{GFP/+}$ mice. (d, e, f);Wfdc2$^{GFP/LacZ}$ mice. Yellow arrowheads indicate GFP$^{neg}$/Hopx$^{pos}$/ProSP-C$^{neg}$ cells (AECIs). Yellow dotted areas in (a) and (d) are enlarged in (b, c) and (e, f), respectively. The scale bar represents 20 µm in (a, d) and 10 µm in (b, c, e, f).

**(E)** AECIs are fairly negative for Wfdc2 both in Wfdc2$^{GFP/+}$ (G/+) and Wfdc2$^{GFP/LacZ}$ (G/L) mice. Quantification of GFP$^{pos}$/Hpox$^{pos}$/ProSP-C$^{neg}$ cells among total Hpox$^{pos}$/ProSP-C$^{neg}$ cells in littermates of Wfdc2$^{GFP/+}$ and Wfdc2$^{GFP/LacZ}$ are shown. Almost all of the Hpox$^{pos}$/ProSP-C$^{neg}$ cells (AECIs) are negative for GFP.

Disease Models & Mechanisms • Supplementary information

**Fig. S5**

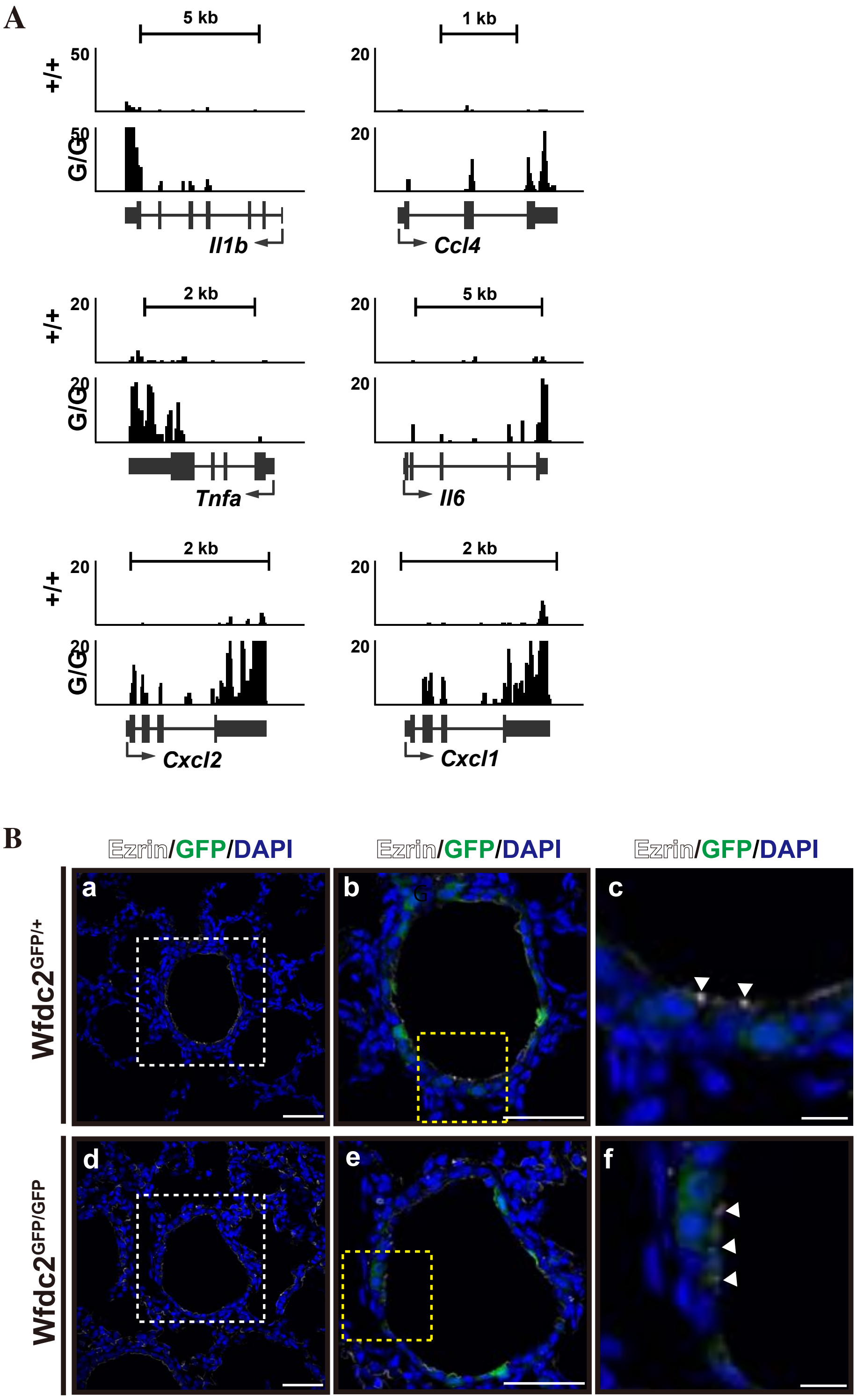


**Fig. S5**

**Up-regulation of acute inflammtory response genes in Wfdc2 deficient mice and IHC analyses of a tight junction protein, ezrin**

**(A)** RNA-seq snapshots of acute inflammatory response genes upregulated in Wfdc2 deficient mice at P1.5. +/+;Wfdc2$^{+/+}$ mice. G/ G;Wfdc2$^{GFP/GFP}$ mice.

**(B)** The tight junction protein, Ezrin, is localized normally in Wfdc2$^{GFP/GFP}$ and Wfdc2$^{GFP/+}$ mice. White

dotted areas in (a) and (d) are enlarged in (b) and (e), respectively. Yellow dotted areas in (b) and (e) are enlarged in (c) and (f), respectively. Triangle indicates ezrin signal. Ezrin; white, GFP; green, DAPI; blue. The scale bar represents 50 µm (a, b, d, e) and 10 µm (c, f).

Disease Models & Mechanisms • Supplementary information

## Table S1

Relative protein expression of surfactant proteins, proteases and junction proteins

| Gene name | | Relative protein expression* | No. of KO | No. of Cont |
|---|---|---|---|---|
| SP-A | | 4.07±1.01 | 8 | 6 |
| SP-D | | 2.06±0.42 | 6 | 6 |
| SP-B | pro | 1.25±0.17 | 6 | 6 |
| | mat | 1.84±0.40 | 7 | 6 |
| SP-C | pro | 1.12±0.07 | 7 | 6 |
| | mat | 1.21±0.18 | 5 | 5 |
| PRSS35 | | 0.72±0.04 | 5 | 5 |
| MMP9 | | 1.12±0.13 | 7 | 7 |
| MMP12 | | 0.97±0.07 | 7 | 5 |
| ADAM10 | | 1.25±0.11 | 7 | 5 |
| E-cadherin | | 1.09±0.05 | 8 | 6 |
| Ezrin | | 1.18±0.07 | 8 | 6 |

* Normalized protein epxression in Wfdc2 deficient lung was compared to that in control ($Wfdc2^{GFP/GFP}$/$Wfdc2^{+/+}$). No. of KO: number of $Wfdc2^{GFP/GFP}$ mice. No. of Cont: number of $Wfdc2^{+/+}$ mice.

Disease Models & Mechanisms • Supplementary information

# Table S2

## Primer list

| Genotyping | Name | primer(5'>3') | | Product size(bp) |
|---|---|---|---|---|
| Wt | Y104 | CAGAGGCTCAAGTCCCAGAG | Forward | 226 |
| | Y105 | GGGAGCAGAGACTTGTCAGG | Reverse | |
| KO | Y222 | AGAAGCGCGATCACATGGTC | Forward | 1032 |
| | PR448 | AAGCCACTGCTCTCAGGTCC | Reverse | |
| GFP | GFP1 | TACGGCAAGCTGACCCTGAA | Forward | 538 |
| | GFP2 | TGTGATCGCGCTTCTCGTTG | Reverse | |
| neo | Y102 | AGAGGCTATTCGGCTATGAC | Forward | 544 |
| | Y103 | CACCATGATATTCGGCAAGC | Reverse | |
| qPCR for Wfdc2 | | | | |
| Wfdc2 | Y663 | AGGACCAGTGTCAGGTGGACAG | Forward | 155 |
| | Y664 | AGATGCACAGTCCGGCAGAA | Reverse | |
| GAPDH | Y538 | CAATGTGTCCGTCGTGGATCT | Forward | 81 |
| | Y539 | GCCTGCTTCACCACCTTCTT | Reverse | |
| Generating probes | | | | |
| 5'probe | Y326 | AGGGGAAAGTGAGTTGAGCA | Forward | 702 |
| | Y327 | TTTCACACCGTTGCTTTCTG | Reverse | |
| 3'probe | Y325 | CAGCTCTGCTGTTGGTGAAC | Forward | 747 |
| | Y324 | CAGGCCCCATATACCTTTGA | Reverse | |

Disease Models & Mechanisms • Supplementary information

# Table S3

## Reagents and Resources

| Reagent or Resource | | SOURCE | IDENTIFER |
|---|---|---|---|
| Antibodies | Dilution | | |
| GFP | 1:1000 | abcam | ab13970 |
| Sox2 | 1:200 | R&D Systems | AF2018 |
| Sox9 | 1:200 | R&D Systems | AF3075 |
| Keratin5 | 1:400 | BioLegend | 905501 |
| CGRP | 1:400 | abcam | ab36001 |
| CC10 | 1:100 | Santa Cruz Biotechnology | SC-9772 |
| FoxJ1 | 1:200 | SIGMA ALDRICH | HPA005714 |
| proSP-C | IHC 1:1250, WB 1:500 | Merck Millipore | AB3786 |
| Podoplanin | 1:1500 | Angio Bio | 11-033 |
| Acetylated tubulin | 1:12000 | SIGMA ALDRICH | T7451 |
| NCS-1 | 1:100 | abcam | ab116230 |
| Hop(E1) | 1:50 | Santa Cruz Biotechnology | sc-398703 |
| E-Cadherin | IHC 1:3000, WB 1:1000 | R&D Systems | AF748 |
| Laminin | 1:200 | Bethesda Research Lab (Gaithersburg, MD) | |
| ABCA3 | 1:200 | abcam | ab24751 |
| IL1 $\beta$ | 1:3600 | abcam | ab9772 |
| CXCL2/MIP-2 | 1:100 | R&D Systems | AF452 |
| SP-A | 1:500 | abcam | ab115791 |
| proSP-B | 1:5000 | SEVEN HILLS | WRAB-55522 |
| matureSP-B | 1:5000 | SEVEN HILLS | WRAB-48604 |
| matureSP-C | 1:5000 | SEVEN HILLS | WRAB-76694 |
| SP-D | 1:1000 | abcam | ab15696 |
| MMP9 | 1:3200 | R&D Systems | AF909 |
| MMP12 | 1:5000 | abcam | ab52897 |
| PRSS35 | 1:1000 | Thermo Fisher Scientific | PA5-32083 |
| ADAM10 | 1:500 | Merck Millipore | AB19026 |
| Ezrin | 1:500 | abcam | ab41672 |
| beta-Actin | 1:5000 | abcam | ab6276 |
| alpha-Tubulin（DM1A) | 1:2000 | SIGMA ALDRICH | T9026 |
| beta-Catenin | 1:2000 | BD transduction laboratories | 610153 |
| Alexa488 anti-chicken IgY | 1:200 | Jackson ImmunoResearch | 703-545-155 |
| Alexa488 anti-rabbit IgG | 1:200 | Thermo Fisher Scientific | A21206 |
| Alexa546 anti-goat IgG | 1:200 | Thermo Fisher Scientific | A11056 |
| Alexa546 anti-mouse IgG | 1:200 | Thermo Fisher Scientific | A10036 |
| Alexa546 anti-rabbit IgG | 1:200 | Thermo Fisher Scientific | A10040 |
| Alexa647 anti-humster IgG | 1:200 | Thermo Fisher Scientific | A21451 |
| Alexa647 anti-rabbit IgG | 1:200 | Thermo Fisher Scientific | A31573 |
| Biotin anti-rabbit IgG | 1:200 | Jackson ImmunoResearch | 7111-066-152 |
| anti-goat IgG H&L-HRP | 1:5000 | abcam | ab97110 |
| anti-mouse IgG H&L-HRP | 1:5000 | abcam | ab6728 |
| anti-rabbit IgG H&L-HRP | 1:5000 | abcam | ab97051 |

| Chemicals, Reagents, and | | | |
|---|---|---|---|
| Skim milk | | Morinaga Milk | |
| 5-20% ePAGEL | | ATO | E-R520L |
| 10-20% ePAGEL | | ATO | E-R1020L |
| 40%acrylamide-bis(29:1) | | nacalai | 06119-45 |

Disease Models & Mechanisms • Supplementary information

| | | |
|---|---|---|
| Chemi-Lumi One L | nacalai tesque | 07880-54 |
| Chemi-Lumi One Super | nacalai tesque | 02230-30 |
| MaxBlot | MBL | 8455-100 |
| Isogen LS | Nippon Gene | 311-02621 |
| Phase Lock Gel Heavy | 5PRIME | 2302810 |
| RQ RNase Free DNase | Promega | M6101 |
| RNAsin | Promega | N261A |
| Super Script III | Thermo Fisher Scientific | 18080-044 |
| FastStartUniversal SYBR | Roche | 04-673-484-001 |
| Hybound-N+ | GE Healthcare | RPN303B |
| Ready-To-Go DNA Labeling Beads(-dCTP) | GE Healthcare | 27-9240-01 |
| ProbeQuant G-50 Micro Columns | GE Healthcare | 28-9043-08 |
| PerfectHyb Hybridization Solution | TOYOBO | HYB-101 |
| dCTP[alpha-$^{32}$P] | Perkin Elmer | NEG513H |
| Bouin's solution | SIGMA ALDRICH | HT10132 |
| Mayer's Hematoxylin solution | Wako | 131-09665 |
| 0.5% Eosin Y Ethanol solution | Wako | 051-06495 |
| O.C.T. compound | Tissue-Tek | 4583 |
| **Critical Commercial Assay** | | |
| Mouse IL-1beta/UK-1F2 Quantikine ELISA Kit | R&D Systems | BLB00C |
| Mouse CXCL2/MIP-2 Quantikine ELISA Kit | R&D Systems | MM200 |
| Mouse CCL4/MIP-1beta Quantikine ELISA Kit | R&D Systems | MMB00 |
| Mouse TNF-alpha Quantikine ELISA Kit | R&D Systems | MTA00B |
| Phosphatidylglycerol Assay Kit | CELL BIOLABS,INC. | MET-5024 |
| Phosphatidylcholine Assay Kit | abcam | ab83377 |

Disease Models & Mechanisms • Supplementary information

## Table S4
## Summary statistics of RNA-seq

| Stage | Genotype | Raw reads | Reads after trimming | Mapped reads | Mapping rate |
|---|---|---|---|---|---|
| E18.5 | Wfdc2_+/+ | 37,807,478 | 37,530,239 | 35,964,194 | 95.80% |
| E18.5 | Wfdc2_+/+ | 32,995,984 | 32,759,910 | 31,641,756 | 96.60% |
| E18.5 | Wfdc2_GFP/GFP | 36,639,184 | 36,435,126 | 36,158,783 | 99.20% |
| E18.5 | Wfdc2_GFP/GFP | 29,489,448 | 29,407,227 | 28,991,266 | 98.60% |
| P1.5 | Wfdc2_+/+ | 23,955,615 | 23,934,272 | 22,864,716 | 95.50% |
| P1.5 | Wfdc2_+/+ | 20,301,493 | 20,291,911 | 19,682,702 | 97.00% |
| P1.5 | Wfdc2_GFP/GFP | 24,249,811 | 24,177,661 | 23,556,081 | 97.40% |
| P1.5 | Wfdc2_GFP/GFP | 30,682,797 | 30,608,985 | 30,072,232 | 98.20% |

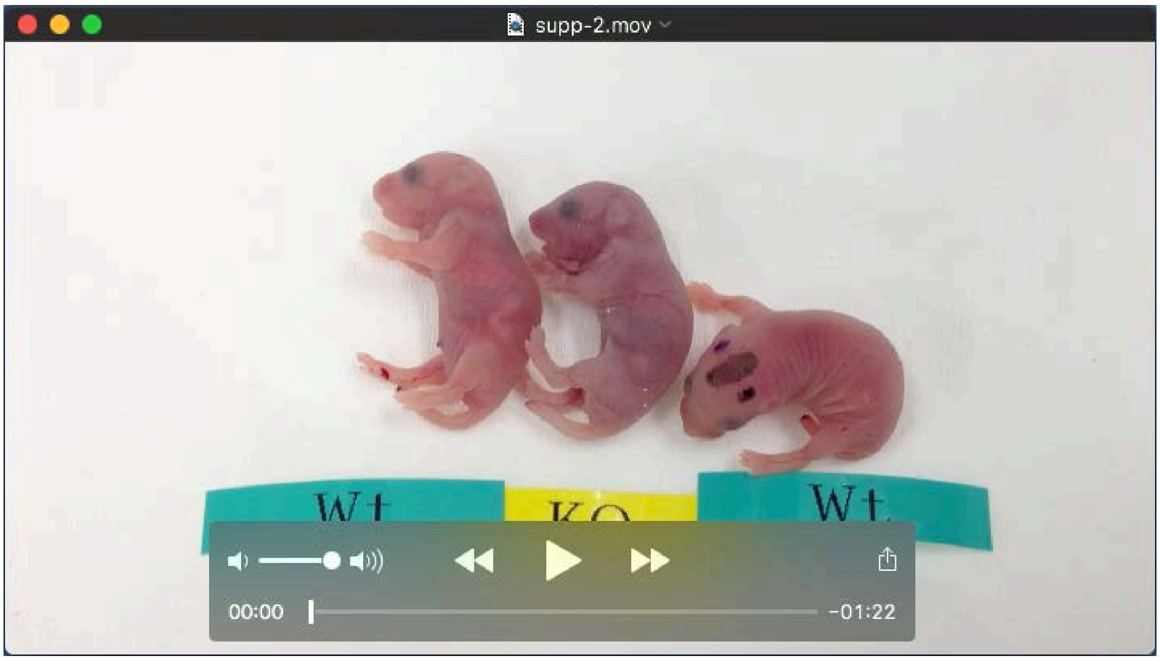


**Movie 1.** *Wfdc2*$^{GFP/GFP}$ neonatal mice became cyanotic immediately after delivery.
